# A novel approach to anxiety level prediction using small sets of judgment and survey variables

**DOI:** 10.1038/s44184-024-00074-x

**Published:** 2024-06-18

**Authors:** Sumra Bari, Byoung-Woo Kim, Nicole L. Vike, Shamal Lalvani, Leandros Stefanopoulos, Nicos Maglaveras, Martin Block, Jeffrey Strawn, Aggelos K. Katsaggelos, Hans C. Breiter

**Affiliations:** 1 Department of Computer Science, University of Cincinnati, Cincinnati, OH USA; 2 Department of Electrical Engineering, Northwestern University, Evanston, IL USA; 3 Laboratory of Medical Informatics, Aristotle University of Thessaloniki, Thessaloniki, Greece; 4 Integrated Marketing Communications, Medill School of Journalism, Northwestern University, Evanston, IL USA; 5 Department of Psychiatry and Behavioral Neuroscience, College of Medicine, University of Cincinnati, Cincinnati, OH USA; 6 Department of Computer Science, Northwestern University, Evanston, IL USA; 7 Department of Radiology, Northwestern University, Chicago, IL USA; 8 Department of Biomedical Engineering, University of Cincinnati, Cincinnati, OH USA; 9 Department of Psychiatry, Massachusetts General Hospital and Harvard School of Medicine, Boston, MA USA

**Keywords:** Computational neuroscience, Machine learning

## Abstract

Anxiety, a condition characterized by intense fear and persistent worry, affects millions each year and, when severe, is distressing and functionally impairing. Numerous machine learning frameworks have been developed and tested to predict features of anxiety and anxiety traits. This study extended these approaches by using a small set of interpretable judgment variables (*n* = 15) and contextual variables (demographics, perceived loneliness, COVID-19 history) to (1) understand the relationships between these variables and (2) develop a framework to predict anxiety levels [derived from the State Trait Anxiety Inventory (STAI)]. This set of 15 judgment variables, including loss aversion and risk aversion, models biases in reward/aversion judgments extracted from an unsupervised, short (2–3 min) picture rating task (using the International Affective Picture System) that can be completed on a smartphone. The study cohort consisted of 3476 de-identified adult participants from across the United States who were recruited using an email survey database. Using a balanced Random Forest approach with these judgment and contextual variables, STAI-derived anxiety levels were predicted with up to 81% accuracy and 0.71 AUC ROC. Normalized Gini scores showed that the most important predictors (age, loneliness, household income, employment status) contributed a total of 29–31% of the cumulative relative importance and up to 61% was contributed by judgment variables. Mediation/moderation statistics revealed that the interactions between judgment and contextual variables appears to be important for accurately predicting anxiety levels. Median shifts in judgment variables described a behavioral profile for individuals with higher anxiety levels that was characterized by less resilience, more avoidance, and more indifference behavior. This study supports the hypothesis that distinct constellations of 15 interpretable judgment variables, along with contextual variables, could yield an efficient and highly scalable system for mental health assessment. These results contribute to our understanding of underlying psychological processes that are necessary to characterize what causes variance in anxiety conditions and its behaviors, which can impact treatment development and efficacy.

## Introduction

Anxiety disorders affected ~12% of the US population in 2021^1^ and affects 4% of the population worldwide^2,3^. Anxiety disorders are characterized by intense fear and persistent worry in the absence of a defined threat^4^ and are among the most common causes of disability worldwide. Anxiety disorders begin early in life^5,6^ and increase the risk of subsequent mood disorders, substance misuse, suicidal behavior and economic disadvantage^7^.

The diagnosis of an anxiety disorder involves clinical determination of the severity of symptoms and the presence of specific symptom constellations based on clinical assessment, commonly augmented by surveys and symptom inventories^8^. Recently, automated approaches have been tested for predicting anxiety, as determined by clinical assessment or surveys, with a primary focus on using machine learning (ML) approaches^9^ with large variable sets (e.g., >100)^10–12^ including clinical data^13–15^, questionnaires^16,17^, wearable biosensors^18–20^, social media posts^21–23^, neural measures from MRI^24–26^ and cognitive science variables^27–31^. These large variable sets add multiple dimensions to the characterization of anxiety across study participants producing higher accuracies and lower unexplained variance. They model complex relationships between the predictors and the outcome, yet can present challenges ranging from significant computational requirements and prohibitive privacy concerns, to lengthy and costly data acquisitions. The current study sought to contribute to current ML-based anxiety level prediction efforts by using a small set of cognitive science variables, that can be acquired in 2–3 min on a small digital device, like a smartphone. Currently, 92% of the US population^32^ and 85% of the world population^33^ can access such devices.

Cognitive science studies focused on judgment behavior are hypothesized to be relevant to anxiety given the overlap in the neural systems implicated in both^34–36^. Abnormalities in reward/aversion judgment have been linked to dopamine system dysfunction in depression, addiction, suicidality, and chronic stress^37–39^, and individuals with anxiety have shown salient alterations in reward/aversion judgment^40–42^. A number of reward/aversion variables are thought to represent biases in judgment^43,44^, such as loss aversion (LA)^45^ and risk aversion (RA)^46^. Heightened RA^47–49^ and heightened LA^50,51^ have been reported in those with anxiety using a range of distinct monetary and emotional stimuli that describe reward/aversion judgment.

Reward/aversion judgment has been studied using operant keypress tasks to frame reinforcement reward in humans^52–56^, and been used to quantify judgment variables like LA^56,57^. These cognitive science studies have compared keypress-based LA to other LA frameworks such as prospect theory (e.g., Lee et al.^58^), connected keypress methods to imaging of reward/aversion circuitry (e.g., refs. ^52,59–61^) and connected LA from operant keypressing to reward/aversion circuitry^62^. The keypress framework allows the modeling of human behavior using variance and entropic variables to produce a set of at least 15 features that characterize an individual’s reward/aversion judgment (e.g., LA, RA, and others, Table 1). These features have been linked to brain structure differences in the context of (1) substance use disorder^60^ and (2) the characterization of substance use disorder and depression^63^.

**Table 1:** Judgment variable abbreviations and descriptions

Judgment variable	Abbreviation	Function/Curve for derivation	Description
Loss aversion	LA	Value function/(*K,H*) curve	The degree to which one overweighs negative stimuli to positive stimuli.
Risk aversion	RA	Value function/(*K,H*) curve	The degree to which one prefers an uncertain high value outcome to something certain but lower value.
Loss resilience	LR	Value function/(*K,H*) curve	Measures ones preference to accept a certain loss vs. uncertain loss. It’s like RA but in the domain of losses.
Ante	Ante	Value function/(*K,H*) curve	The degree one is willing to pay to enter a game of chance (e.g., poker).
Insurance	Insurance	Value function/(*K,H*) curve	The amount of security one is willing to acquire to avoid negative outcomes.
Peak positive risk	Peak PR	Limit function/(*K,σ*) curve	Per Markowitz’s decision utility equation, this is the peak risk around approach choices that must be overcome for approach behavior to occur.
Peak negative risk	Peak NR	Limit function/(*K,σ*) curve	Per Markowitz’s decision utility equation, this is the peak risk around avoidance choices that must be overcome for avoidance behavior to occur.
Reward tipping point	Reward TP	Limit function/(*K,σ*) curve	Per Markowitz’s decision utility equation, this is the reward value beyond which approach choices are made.
Aversion tipping point	Aversion TP	Limit function/(*K,σ*) curve	Per Markowitz’s decision utility equation, this is the intensity of aversion beyond which avoidance choices are made.
Total reward risk	Total RR	Limit function/(*K,σ*) curve	Total value of reward across the range of risks associated with positive outcomes.
Total aversion risk	Total AR	Limit function/(*K,σ*) curve	Total amount of aversion across the range of risks associated with negative outcomes.
Reward-aversion tradeoff	RA tradeoff	Value function/(*H*_*+*_*,H*_−_) curve	This represents the average bias of information towards approach or avoidance behavior.
Tradeoff range	Tradeoff range	Value function/(*H*_*+*_*,H*_−_) curve	The variance or bias towards approach versus avoidance behavior, and is one metric of the range in a person’s portfolio of preferences.
Reward-aversion consistency	RA consistency	Value function/(*H*_*+*_*,H*_−_) curve	A continuum between how much an individual has conflict in their reward-aversion preference versus indifference in their preference—where conflict means they both like and dislike something while indifference means they don’t like or dislike something.
Consistency range	Consistency range	Value function/(*H*_*+*_*,H*_−_) curve	How much a person swings between conflict and indifference in their preferences; it is a second metric regarding the range in a person’s portfolio of preference.

The value function curve derived from (*K,H*) variables provides five preference variables: LA, RA, LR, Ante and Insurance. The limit function curve, derived from (*K,σ*) variables, provides six preference variables: Peak PR, Peak NR, Reward TP, Aversion TP, Total RR and Total AR. The tradeoff function, derived from (*H*_*+*_*,H*_−_) variables, provides four preference variables: RA tradeoff, Tradeoff range, RA consistency and Consistency range. The four columns represent the full term for each of the 15 judgment variables, their abbreviations as used in this manuscript, the equation on which they are based, and their description. To read about Markowitz’s decision utility equation, please see ref. ^113^.

**Fig. 1:**
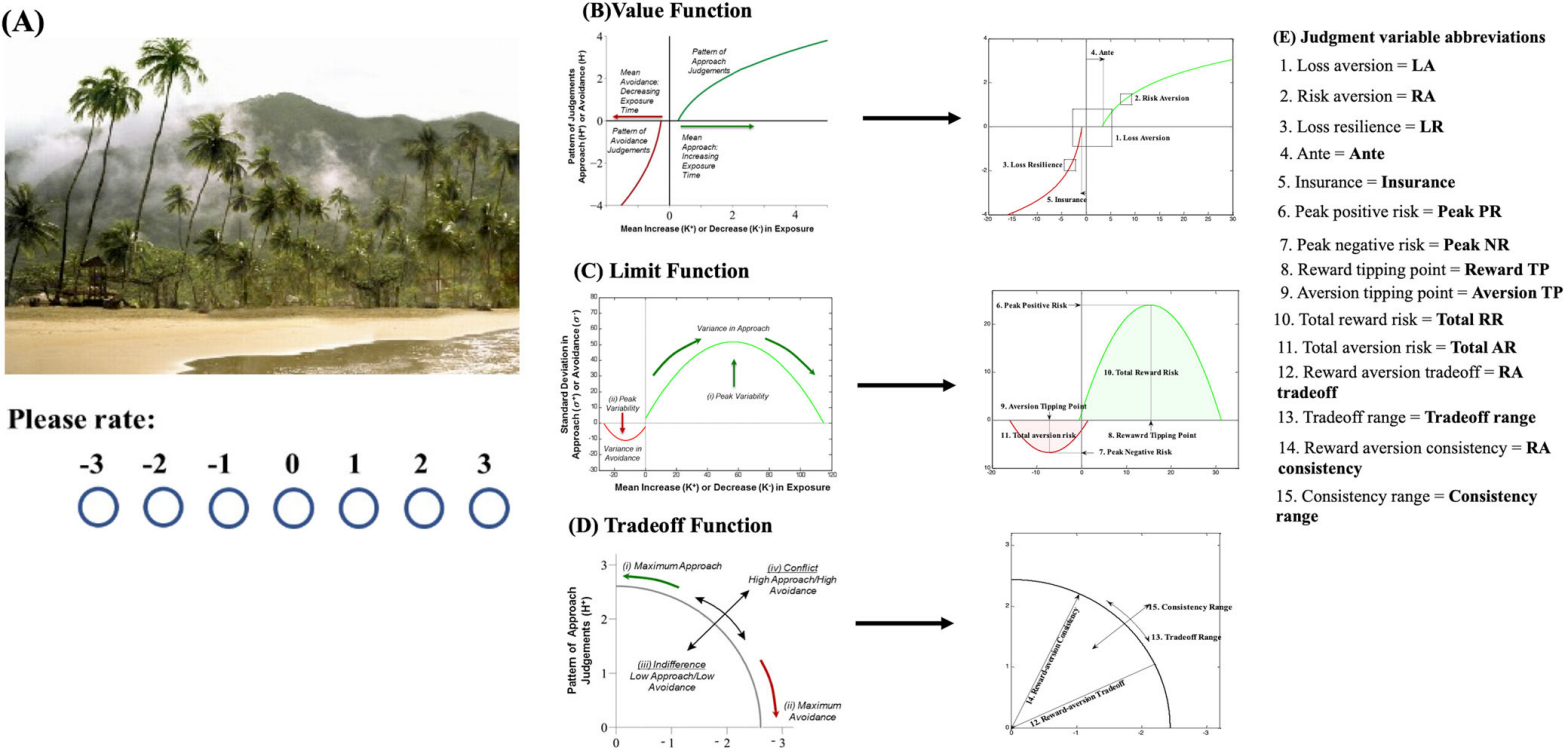
Picture rating task and judgment variable extraction. **A** An example picture from the picture rating task where participants were asked to rate how much they liked or disliked an imagine on a scale of −3 (dislike very much) and +3 (like very much), with 0 being neutral. **B** Visual representation of the x–y plane for relative preference theory (RPT) *value function* fitting and resulting features extracted. **C** Visual representation of the x–y plane for RPT *limit function* fitting and resulting features extracted. **D** Visual representation of the x–y plane for RPT *tradeoff function* fitting and resulting features extracted. **E** Each of the 15 features and their abbreviated terms.

These 15 judgment variables can also be computed from a picture rating task that takes 2–3 min^64^. The picture rating task was adapted from the operant keypress task, and is implementable on a smartphone or digital device (Fig. 1, Table 1). Judgment variables from the shorter picture rating task are consistent across multiple data sets^64,65^. The 15 judgment variables derived from the picture rating task, when combined with a small set of demographic and survey variables, have been used to predict other mental health and medical health conditions with high accuracy using ML: depression history^65^, suicidality^66^, and vaccine uptake^67^. Based on results from these prior publications, we hypothesized that a small set of 15 judgment variables (see Fig. 1, Table 1), with contextual variables theorized to affect judgment and mental function (in this case: demographics, perceived loneliness, and COVID-19 history), might facilitate the prediction of anxiety levels.

A short picture rating task (see “Methods”) was administered to 4019 (3476 following data exclusion) de-identified participants in December 2021. Participants rated 48 unique color images from the International Affective Picture System (IAPS)^68,69^ on a scale of −3 (dislike very much) to +3 (like very much). Anxiety scores were derived from the state component of the State-Trait Anxiety Inventory (STAI) questionnaire^70^, a validated anxiety questionnaire. Random Forest (*RF*) and balanced Random Forest (*bRF*) techniques were used to classify anxiety scores into ‘higher’ versus ‘lower’ classes and to understand the relative importance of the predictors using Gini scores. Post hoc mediation/moderation analyses were conducted to understand the interactions between judgment and contextual variables that may underly anxiety level prediction. Lastly, contextual and judgment variable differences were assessed against anxiety levels.

This study took the perspective that the power of psychological constructs depends on their capacity to make meaningful predictions. The use of mathematical cognitive science to predict survey-based anxiety measures contributes to our understanding of how psychological processes underlie the variance in anxiety conditions and behaviors, which might impact treatment development and efficacy.

## Results

This study assessed anxiety levels (derived from STAI questionnaire) in relation to contextual and 15 picture rating-derived judgment variables. Judgment variables include Loss Aversion (LA)^45^, Risk Aversion (RA)^46^, Loss Resilience (LR), Ante, Insurance, Total Reward Risk (Total RR), Total Aversion Risk (Total AR), Peak Positive Risk (Peak PR), Peak Negative Risk (Peak NR), Reward Tipping Point (Reward TP), Aversion Tipping Point (Aversion TP), Reward-Aversion tradeoff (RA tradeoff), Tradeoff range, Reward-Aversion consistency (RA consistency) and Consistency range (see Fig. 1B–D and Table 1).

### Classification analysis: Random Forest (*RF*) and balanced Random Forest (*bRF*)

For the classification of ‘higher’ and ‘lower’ anxiety levels, the *bRF* performed better than *RF* in terms of sensitivity, specificity, AUC ROC, and balanced accuracy at all three threshold values with the best performance for the threshold of 35 (Table 2). For the *bRF* classification, the out of bag (OOB) accuracy and accuracy ranged from 72 to 81% and AUC ROC ranged from 0.71 to 0.74 (Table 2) which was much higher than the chance levels obtained from the permutation analysis (Supplementary Table 3). The sensitivity ranged from 56 to 74% with the lowest values corresponding to a greater class imbalance at a threshold of 55. A greater class imbalance was noted as the threshold values increased as depicted in the ‘Percentage of data’ column of Table 2. When the threshold value was 55, the percentage of participants with high anxiety was only 12% (418/3476) of the dataset.

**Table 2:** Random Forest (*RF*) and balanced Random Forest (*bRF*) results for prediction of anxiety/STAI-S scores in to ‘higher (H)’ and ‘lower (L)’ classes

STAI-S threshold	Percentage of data		Classifier	OOB accuracy	Accuracy	Sensitivity	Specificity	AUC ROC	Balanced accuracy
	Class ‘H'	Class ‘L'							
35	50.09	49.91	*RF*	71.85	71.70	73.95	69.49	0.7172	71.72
			*bRF*	72.10	72.36	74.14	70.62	0.7238	72.38
45	30.44	69.56	*RF*	77.01	77.30	46.98	90.24	0.6877	68.61
			*bRF*	76.60	75.12	69.52	77.51	0.7352	73.52
55	12.02	87.97	*RF*	88.24	88.51	3.25	99.78	0.5152	51.52
			*bRF*	81.30	81.29	56.10	84.62	0.7077	70.36

The predictors used were 15 judgment variables (see Fig. 1 and Table 1), eleven contextual variables including: loneliness, age, sex, income, marital, employment, edu, edu_years race/ethnicity, test and diagnosis (see “Methods” and Supplemental Material for details).

*OOB* out of bag, *AUC ROC* area under the receiving operating characteristics curve.

For *RF* classification, the OOB accuracy and test dataset accuracy ranged from 72 to 88% and AUC ROC ranged from 0.52 to 0.72 (Table 2). The sensitivity ranged from 3 to 74% with the lowest values corresponding to a greater class imbalance at a threshold of 55. *RF* had the worst performance at the threshold of 55, with the output metrics close to the chance levels (Supplementary Table 3).

Multi-dimensional scaling (MDS) plots demonstrated how ‘higher’ and ‘lower’ clusters were better distinguished at lower STAI-S thresholds (i.e., Supplementary Fig. 2) and how *bRF* always produced better data segregation between the two classes as compared to *RF*.

### Relative importance of features

**Fig. 2:**
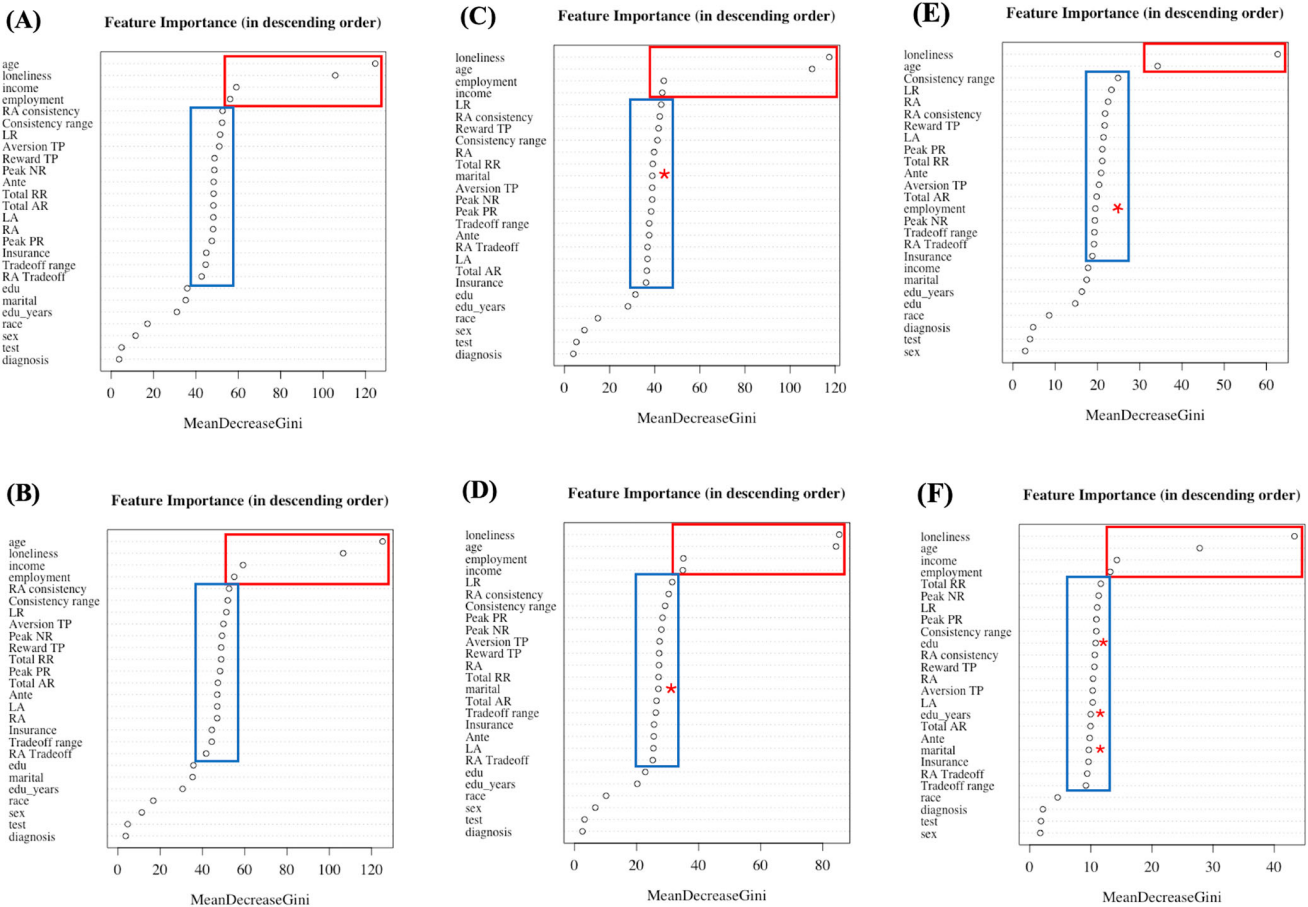
Gini score plots for Random Forest (*RF*) and balanced Random Forest (*bRF*) analyses. The predictors are arranged according to the mean decrease in Gini scores with the most important predictors on the top. The red box outlines the top contextual variables, and the blue box outlines the 15 judgment variables while the red * in the blue box points to the contextual variables in the cluster of judgment variables. Plots (**A**), (**C**), (**E**) corresponds to *RF* analyses with thresholds of 35, 45, and 55, respectively, and (**B**), (**D**), (**F**) corresponds to *bRF* with thresholds of 35, 45, and 55, respectively.

Age, loneliness, income, and employment were consistently the most important individual features for *bRF* based on the mean decrease in Gini scores (see Fig. 2B, D, F). This was also the case for *RF* with anxiety thresholds of 35 and 45 (Fig. 2A, C). Together, these four variables contributed between 29 and 33% of the relative importance (Table 3). For the *RF* analysis with a high threshold of 55 (only 12% of the cohort in the ‘higher’ group and 3% sensitivity), age and loneliness remained the top-most contributing variables.

**Table 3:** The relative importance of the features based on normalized Gini scores from Random Forest (*RF*) and balanced Random Forest (*bRF*) analyses

Feature	Relative importance
A	
Age	0.1029
Loneliness	0.0873
Income	0.0488
Employment	0.0464
RA consistency	0.0434
Consistency range	0.0433
LR	0.0425
Aversion TP	0.0421
Reward TP	0.0403
Peak NR	0.0402
Ante	0.0399
Total RR	0.0399
Total AR	0.0398
LA	0.0398
RA	0.0397
Peak PR	0.0393
Insurance	0.0371
Tradeoff range	0.0368
RA Tradeoff	0.0353
edu	0.0297
Marital	0.0291
edu_years	0.0256
Race	0.0142
Sex	0.0095
Test	0.0041
Diagnosis	0.0031
B	
Age	0.1040
Loneliness	0.0885
Income	0.0492
Employment	0.0457
RA consistency	0.0437
Consistency range	0.0432
LR	0.0426
Aversion TP	0.0415
Peak NR	0.0409
Reward TP	0.0406
Total RR	0.0406
Peak PR	0.0401
Total AR	0.0393
Ante	0.0390
LA	0.0390
RA	0.0390
Insurance	0.0369
Tradeoff range	0.0369
RA Tradeoff	0.0347
edu	0.0297
Marital	0.0294
edu_years	0.0254
Race	0.0139
Sex	0.0094
Test	0.0038
Diagnosis	0.0031
C	
Loneliness	0.1140
Age	0.1066
Employment	0.0427
Income	0.0421
LR	0.0416
RA consistency	0.0410
Reward TP	0.0405
Consistency range	0.0400
RA	0.0386
Total RR	0.0380
Marital	0.0378
Aversion TP	0.0378
Peak NR	0.0377
Peak PR	0.0373
Tradeoff range	0.0365
Ante	0.0364
RA Tradeoff	0.0357
LA	0.0357
Total AR	0.0354
Insurance	0.0351
edu	0.0305
edu_years	0.0273
Race	0.0143
Sex	0.0086
Test	0.0051
Diagnosis	0.0038
D	
Loneliness	0.1149
Age	0.1135
Employment	0.0472
Income	0.0470
LR	0.0424
RA consistency	0.0408
Consistency range	0.0393
Peak PR	0.0382
Peak NR	0.0377
Aversion TP	0.0369
Reward TP	0.0367
RA	0.0366
Total RR	0.0364
Marital	0.0364
Total AR	0.0356
Tradeoff range	0.0352
Insurance	0.0345
Ante	0.0344
LA	0.0342
RA Tradeoff	0.0339
edu	0.0307
edu_years	0.0272
Race	0.0137
Sex	0.0090
Test	0.0043
Diagnosis	0.0034
E	
Loneliness	0.1209
Age	0.0660
Consistency range	0.0479
LR	0.0449
RA	0.0434
RA consistency	0.0421
Reward TP	0.0419
LA	0.0413
Peak PR	0.0408
Total RR	0.0408
Ante	0.0402
Aversion TP	0.0393
Total AR	0.0382
Employment	0.0376
Peak NR	0.0374
Tradeoff range	0.0372
RA Tradeoff	0.0370
Insurance	0.0363
Income	0.0343
Marital	0.0337
edu_years	0.0315
edu	0.0284
Race	0.0165
Diagnosis	0.0092
Test	0.0078
Sex	0.0056
F	
Loneliness	0.1468
Age	0.0943
Income	0.0483
Employment	0.0446
Total RR	0.0394
Peak NR	0.0382
LR	0.0374
Peak PR	0.0370
Consistency range	0.0369
edu	0.0365
RA consistency	0.0361
Reward TP	0.0358
RA	0.0351
Aversion TP	0.0349
LA	0.0349
edu_years	0.0338
Total AR	0.0337
Ante	0.0332
Marital	0.0328
Insurance	0.0326
RA Tradeoff	0.0319
Tradeoff range	0.0311
Race	0.0154
Diagnosis	0.0073
Test	0.0062
Sex	0.0058

Tables (A), (C), (E) corresponds to *RF* analyses with thresholds of 35, 45 and 55, respectively, and (B), (D), (F) corresponds to *bRF* with thresholds of 35, 45 and 55, respectively.

The 15 judgment variables contributed a combined 55–61% of the relative importance (Fig. 2 and Table 3). Other contextual variables (education group, education in years, marital status, race/ethnicity, sex, COVID-19 test, and diagnosis) were lower in classification importance, contributing a combined relative importance of 11–21%.

### Mediation and moderation analysis

Mediation and moderation analysis were used to define statistical interactions between judgment variables and the most important contextual variables (age, loneliness, income, and employment). These contextual variables were defined as the mediator (Me) or moderator (Mo), judgment variables were defined as the independent variable, and STAI-S scores were defined as the dependent variable. These analyses revealed nine mediation (Table 4A and Supplementary Table 4) and seven moderation results (Table 4B). Age acted as a mediator when loss resilience, Aversion TP, Tradeoff Range, and Consistency range were independent variables. Loneliness appeared as the mediator in four mediation analyses with ante, insurance, Total AR, and Consistency range, whereas employment only mediated Consistency range as the independent variable.

**Table 4:** Mediation and moderation results

A	
Mediator (Me)	Independent variable (X)
Age	Loss resilience
	Aversion TP
	Tradeoff range
	Consistency range
Loneliness	Ante
	Insurance
	Total AR
	Consistency range
Employment	Consistency range
Income	–

B			
Moderator (Mo)	Independent variable (X)	*pβ*_3_	*p*_*overall*_
Age	Ante	0.001	<0.0001
	Insurance	0.014	<0.0001
	Peak PR	0.010	<0.0001
	Peak NR	0.008	<0.0001
Loneliness	Peak PR	0.042	<0.0001
Employment	Insurance	0.001	<0.0001
	Peak NR	0.028	<0.0001
Income	–	–	–

(A) Significant results for Mediation analyses. The four most important contextual variables (age, loneliness, employment and income) from (Fig. 2 and Table 3) acted as mediator (Me) and the 15 judgment variables were independent variable (X) to model anxiety score (Y). For detailed mediation results with all beta coefficients and *p*-values see Supplemental Table 4. ‘–’ indicates no significant results were obtained with contextual variable income. (B) Significant results for Moderation analyses. The four most important contextual variables (age, loneliness, employment and income) from (Fig. 2 and Table 3) acted as moderator (Mo) and the 15 judgment variables were independent variables (X) to model anxiety score (Y). Moderation was considered significant if ${p}_{{\beta }_{3}}\le 0.05$ (the interaction term ${\beta }_{3}$ is significantly different than zero) and ${p}_{{overall}}\le 0.05$ (for the overall model). ‘–’ indicates no significant results were obtained with contextual variable income.

Independent and Me variables were then switched to test if the judgment variables acted as mediators. No significant mediation results were found when contextual variables were independent variables and judgment variables were mediators.

With regard to moderation analyses, age was found to also be involved in four moderations, with ante, insurance, Peak PR, and Peak NR as the independent variables. Loneliness was involved in one moderation with Peak PR, and employment was implicated in two moderations with insurance and Peak NR. There were no mediation or moderation results with the income variable.

Note that age, loneliness, and employment interacted with different judgment variables when acting as a mediators versus when acting as a moderators (see Table 4A and Table 4B).

### Post hoc analysis of contextual variable differences

The seven demographic variables (excluding years of education), perceived loneliness, and COVID-19 history were assessed for differences across anxiety scores using Wilcoxon rank-sum and Kruskal Wallis tests. All contextual variables significantly varied by anxiety score (*p*-value < 0.05) (Table 5A). For the majority of contextual variables, boxplots depicted ascending or descending trends (Supplementary Fig. 3). For example, anxiety scores were higher (1) with higher levels of perceived loneliness, (2) among younger individuals, (3) in females, (4) among individuals with lower household income, (5) among individuals with lower education levels, and (6) in individuals reporting a history of COVID-19 infection (test and diagnosis).

**Table 5:** (A) Anxiety score differences by levels of contextual variables and (B) Location shift in the medians for ‘higher’ and ‘lower’ groups of judgment variables

A		
Variable	Test	*p*-value
Loneliness	Kruskal Wallis	<2.2E−16
Age (3 levels)	Kruskal Wallis	<2.2E−16
Sex	Kruskal Wallis	<2.2E−16
Income	Kruskal Wallis	<2.2E−16
Marital	Kruskal Wallis	<2.2E−16
Employment	Kruskal Wallis	<2.2E−16
Edu	Kruskal Wallis	<2.2E−16
Race	Kruskal Wallis	1.25E−06
Test	Wilcoxon rank sum	1.31E−12
Diagnosis	Wilcoxon rank sum	3.24E−09

B			
RPT	STAI-S threshold	Alternative hypothesis	*p*-value
Loss aversion	35	***H*** > ***L***	***0.029***
		H < L	0.971
	45	H > L	0.055
		H < L	0.946
	55	H > L	0.151
		H < L	0.849
Risk aversion	35	H > L	0.944
		H < L	0.056
	45	H > L	0.987
		***H*** < ***L***	***0.013***
	55	H > L	0.945
		H < L	0.055
Loss resilience	35	H > L	0.999
		***H*** < ***L***	0.001*
	45	H > L	1.000
		***H*** < ***L***	9.007E-07*
	55	H > L	1.000
		***H*** < ***L***	1.98E-04*
Ante	35	H > L	0.162
		H < L	0.839
	45	***H*** > ***L***	***0.022***
		H < L	0.978
	55	***H*** > ***L***	***0.041***
		H < L	0.959
Insurance	35	H > L	0.122
		H < L	0.878
	45	H > L	0.874
		H < L	0.126
	55	H > L	0.914
		H < L	0.086
Peak PR	35	H > L	0.478
		H < L	0.522
	45	***H*** > ***L***	0.002*
		H < L	0.998
	55	***H*** > ***L***	***0.008***
		H < L	0.992
Peak NR	35	H > L	0.998
		***H*** < ***L***	0.002*
	45	H > L	0.914
		H < L	0.086
	55	H > L	0.112
		H < L	0.888
Reward TP	35	H > L	0.461
		H < L	0.539
	45	H > L	0.602
		H < L	0.398
	55	H > L	0.335
		H < L	0.665
Aversion TP	35	H > L	0.999
		***H*** < ***L***	0.001*
	45	H > L	0.999
		***H*** < ***L***	0.001*
	55	H > L	0.933
		H < L	0.067
Total RR	35	***H*** > ***L***	***0.049***
		H < L	0.951
	45	***H*** > ***L***	1.397E-04*
		H < L	1.000
	55	***H*** > ***L***	0.004*
		H < L	0.996
Total AR	35	H > L	0.999
		***H*** < ***L***	0.001*
	45	H > L	0.933
		H < L	0.067
	55	H > L	0.241
		H < L	0.759
RA Tradeoff	35	H > L	0.809
		H < L	0.191
	45	H > L	0.914
		H < L	0.086
	55	H > L	0.398
		H < L	0.602
Tradeoff range	35	H > L	1.000
		***H*** < ***L***	4.716E-11*
	45	H > L	1.000
		***H*** < ***L***	1.333E-14*
	55	H > L	1.000
		***H*** < ***L***	2.153E-04*
RA consistency	35	H > L	0.991
		***H*** < ***L***	***0.009***
	45	H > L	1.000
		***H*** < ***L***	2.603E-04*
	55	H > L	0.509
		H < L	0.491
Consistency range	35	H > L	0.939
		H < L	0.061
	45	H > L	0.629
		H < L	0.371
	55	H > L	0.449
		H < L	0.552

Kruskal Wallis and Wilcoxon rank-sum test results with a significance of α < 0.05. The judgment variable values were divided into ‘higher’ and ‘lower’ groups corresponding to anxiety score groups based on the thresholds of 35, 45, and 55. One-sided Wilcoxon rank sum test results with a significance of *α* < 0.05 are presented in bold. ‘*’ indicates the *p*-values that were significant with Bonferroni correction (*α* < 0.0083), where correction was done across all six tests for each judgment variable. The alternative hypothesis indicated if the distribution median for the judgment variable in the ‘higher (H)’ anxiety group was greater than the ‘lower (L)’ anxiety group or vice versa.

### Post hoc analysis of judgment variable differences

**Fig. 3:**
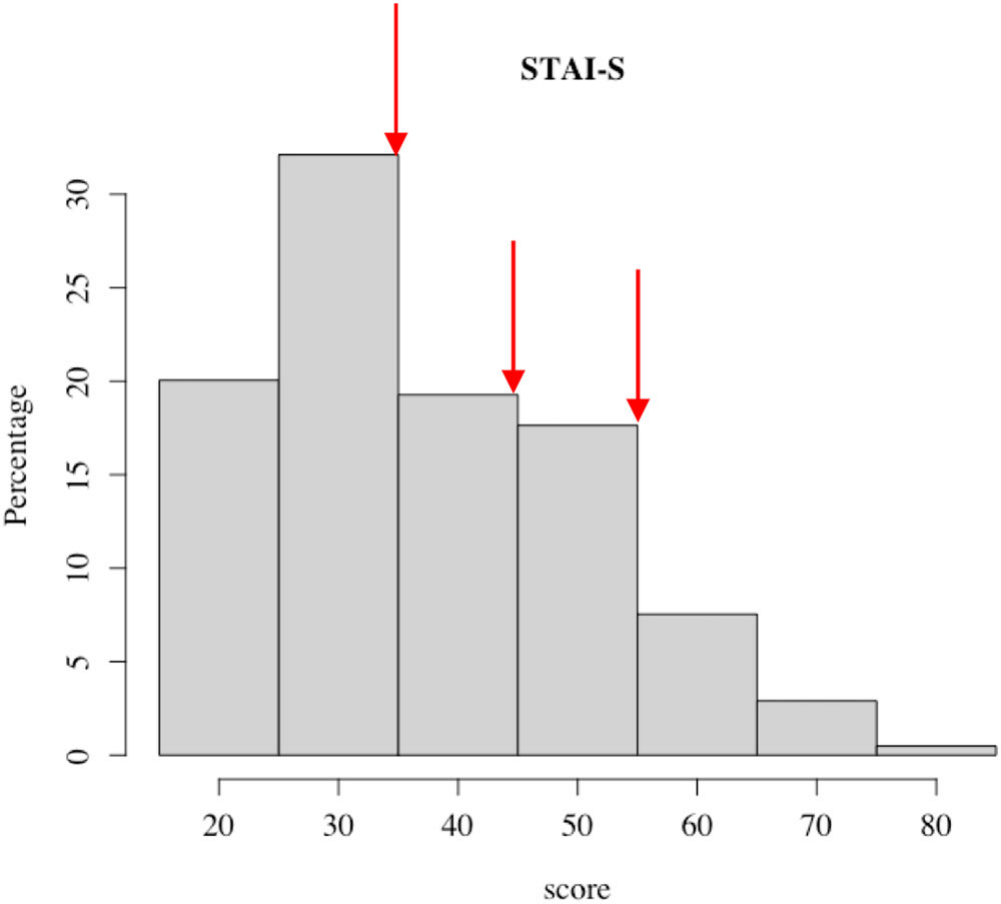
Histogram of anxiety/STAI-S scores with red arrows indicating the threshold values of 35, 45, and 55 for dividing anxiety scores into ‘lower’ and ‘higher’ groups for classification analyses. The thresholds 35, 45, and 55 roughly corresponds to 50th percentile (median), 75th percentile, and 90th percentile respectively of the anxiety/STAI-S scores. All values below the threshold are considered in ‘lower’ group and values above and equal to threshold are in ‘higher’ group.

Judgment variables were analyzed by ‘higher’ and ‘lower’ anxiety scores for the three threshold values (Fig. 3). Eleven out of the 15 judgment variables differed using the one-sided Wilcoxon rank sum test (significance *α* < 0.05) and 8 out 15 differed after correction for multiple comparisons (significance *α* < 0.0083, after Bonferroni correction). The alternative hypothesis, and the respective *p*-values for each test are reported in Table 5B. The alternative hypothesis was defined as the judgment variable distribution median being greater in the ‘higher’ anxiety group than the ‘lower’ anxiety group, or vice versa. The ‘higher’ anxiety group had *higher* medians for loss aversion (threshold = 35, *p* < 0.05), ante (threshold = 45, 55, *p* < 0.05), Peak PR (threshold = 45, *p* < 0.0083; 55, *p* < 0.05), and Total RR (threshold = 35, *p* < 0.05; 45, 55, *p* < 0.0083) when compared to the ‘lower’ anxiety group (Table 5B). The ‘higher’ anxiety group had *lower* medians for risk aversion (threshold = 45, *p* < 0.05), loss resilience (threshold = 35, 45, 55, *p* < 0.0083), Peak NR (threshold = 35, *p* < 0.0083), Aversion TP (threshold = 35, 45, *p* < 0.0083), Total AR (threshold = 35, *p* < 0.0083), Tradeoff Range (threshold = 35, 45, 55, *p* < 0.0083), and RA consistency (threshold = 35, *p* < 0.05; 45, *p* < 0.0083) (Table 5B). Insurance, Reward TP, RA Tradeoff, and Consistency Range showed no significant differences across all threshold values.

## Discussion

This study evaluated how well a small set of judgment and contextual variables (i.e., demographics, perceived loneliness, and COVID-19 infection history), could together predict state anxiety levels. The study produced four major findings. First, prediction accuracy ranged from 72.4 to 81.3% with balanced Random Forest (*bRF)*, and sensitivities decreased (74.1% to 56.1%) as the threshold for classifying anxiety was increased. Regardless of the threshold change, all prediction models maintained a relatively high AUC ROC (0.72 to 0.71), comparable with findings in the literature, and distinct from a permutation analysis showing AUC ROC outcomes approximating 0.50. *bRF* produced uniformly higher sensitivity outcomes than *RF* approaches. Second, four contextual variables (age, income, employment, and perceived loneliness) had the highest relative importance based on normalized Gini scores across most *RF* and *bRF* analyses (5 out of 6 analyses), and contributed a cumulative of 29–33% of relative importance to prediction. Other contextual variables such as race/ethnicity, sex, and COVID-19 infection history showed minimal importance across all analyses. All 15 judgment variables consistently showed similar importance and contributed a cumulative importance ranging from 55 to 61%. Third, nine of the 15 judgment variables were involved in mediation or moderation with contextual variables. Age, employment, and perceived loneliness mediated or moderated relationships with distinct judgment variables to model anxiety scores, consistent with other reports regarding the relationship of cognitive science measures and contextual variables^65–67,71–75^. Fourth, all contextual variables exhibited significant differences in anxiety scores, and 11 of the 15 judgment variables differed when assessed for median shifts across ‘higher’ and ‘lower’ anxiety groups, indicating that a constellation of judgment alterations are predictive of anxiety levels.

Prediction results from this study were comparable to recent research deploying advanced machine learning algorithms to predict anxiety and other mental health conditions^9^, with a number of limitations and advantages. Over the past decade there have been six general types of data used for predicting anxiety, with considerable heterogeneity in terms of how anxiety is defined, and the machine learning algorithms used. These six general frameworks for prediction included variables from: neuroimaging, physiological signals, survey-based assessments, social media posts, clinical or medical health records and behavioral tasks. Neuroimaging studies targeting anxiety prediction reported high accuracies ranging between 79 and 90%^24,25^ and low correlation r = 0.28 (using Gaussian Process Regression)^26^ but present challenges surrounding the collection of expensive, computationally-intensive, and complex MRI data that requires supervision of trained individuals at specific imaging sites. With similar caveats, studies using bio-signals and physiological signals^18–20^, that involved the use of multiple wearable sensors and collection of data under supervision, reported higher accuracies and model fits of 84.3%, 89.8% and r = 0.81. Survey-based studies have utilized extensive sets of demographic variables and lengthy questionnaires^16,17^, to predict anxiety with sensitivities between 62 and 73%. Other studies using demographics, lifestyle, and health surveys^10,11^, and transcripts of recorded interviews^12^ predicted self-reported anxiety with accuracies between 75–86%. Studies using social media platforms like Reddit predicted anxiety with 75% precision^21^, 78% accuracy^22^) from posts in mental health discussion groups, and Gruda et al.^23^ used tweets to predict anxiety scores based on the level of anxiety assessed by volunteers in those tweets. Some studies^13–15^ required access to clinical and medical records of thousands of participants and reported accuracies of 73–89%^11,13,14,18,20,25^.

A number of studies have utilized cognitive science variables derived from behavioral tasks to study anxiety^27,28,31^ and neuroticism^29^ (a general trait that is considered as a vulnerability factor for anxiety^76^). For instance, Yamamori et al.^27^ used approach-avoidance reinforcement learning tasks with hierarchical logistic regression (reporting *p* < 0.05) to model task-induced anxiety. Aupperle et al.^28^ reported significant correlations (*p* < 0.01) between measures from computer-based approach-avoidance conflict task and self-reported anxiety measures from anxiety sensitivity index and Behavioral Inhibition/Activation Scale. Park et al.^29^ reported attenuated processing of gains and losses (using functional MRI responses to a Monetary Incentive Delay task) with higher polygenic risk scores for neuroticism using a general linear model (*p* < 0.001). Forthman et al.^30^ predicted repetitive negative thinking (a trait that negatively impacts anxiety) from 20 principal components of behavioral and cognitive variables (derived from detailed neuropsychological and behavioral assessment) and polygenic risk scores using a machine learning ensemble method with R^2^ of 0.037 (standard error = 0.002). Richter et al.^31^ utilized thorough behavioral testing completed by participants under supervision, and reported a sensitivity of 71.4% and a specificity of 70.8% using *RF* in individuals with anxiety and/or depression. The current study complements these publications, and supports their findings by using a machine learning-based approach and a short cognitive science task that can be performed without supervision on personal electronic device to predict anxiety with high accuracy and sensitivity.

Four contextual variables (age, loneliness, income, and employment status) were salient for the prediction of anxiety, having a cumulative relative importance of 29–33%. Although the relationship between anxiety levels and demographic measures was consistent with the literature (as described below), most of the other demographic measures did not contribute as much to anxiety level prediction. For instance, sex (gender assigned at birth) consistently contributed less than 1% of relative importance and race/ethnic background contributed 1–1.5% of relative importance across analyses. The 15 judgment variables contributed a cumulative relative importance ranging from 55 to 61%. These variables quantify irrationality, or biases, in judgment (i.e., the bounds to rationality as described by Kahneman^44^), and support prior publications pointing to the importance of reward/aversion variables for the study anxiety^27,28,41,77^. Gini scores were minimally different across the 15 judgment variables, suggesting further research is needed to assess how these judgment variables interact or cluster together. When the threshold used for segregating ‘higher’ versus ‘lower’ anxiety groups was increased, education and marital status increased in feature importance for prediction, raising a hypothesis that some demographic variables may be more important for predicting severe anxiety. A history of COVID-19 infection was not salient for predicting current anxiety and was consistently one of the least important features. This result contrasts with other literature showing large-scale societal concern about COVID-19 illness, the pandemic and related anxiety^78,79^. This study only used the “state” and not the “trait” component from the STAI, to reflect current experience. The study thus does not address any relationship between prior COVID-19 history and long-term trait anxiety, nor address people’s thoughts about infection.

Mediation and moderation models were used to quantify relationships between contextual and judgment variables involved in the prediction of anxiety levels. Three of the four most important contextual variables (age, employment, and loneliness) interacted with judgment variables to predict, or model, anxiety scores in mediation and moderation frameworks. These relationships were not observed with income and no significant mediation were found when contextual variables (age, employment, income, and loneliness) acted as independent variables and judgment variables were the mediator variables. On the other hand, significant mediations with contextual variables as mediators indicated that the contextual variables statistically modulated the relationship between judgment variables and anxiety scores; that is, they sat in the causal pathway between judgment variables and anxiety levels. Seven unique judgment variables were involved in nine significant mediation models with age, employment, and loneliness as mediators. Moderation analyses reflected how an interaction between a contextual variable and judgment variable might predict anxiety scores; these relationships were observed for only four judgment variables across seven significant moderation models. In total, 9 of the 15 judgment variables were thus involved in either mediation or moderation, indicating that contextual variables affect the impact of a majority of the judgment variables on anxiety level prediction. From a psychological perspective, these findings demonstrate how context (e.g., age, employment status, perceived loneliness) modulates or interacts with judgment variables to model anxiety, and how these relationships between judgment and context may aide the assessment of anxiety and ultimately, other mental health conditions. Others have noted that psychological processes occur in a context, and this study supports their work^65–67,71–75^.

In the current study, anxiety scores significantly varied by the contextual variables used to classify anxiety scores. Anxiety scores increased with increasing levels of perceived loneliness, where participants who often, or always, avoid spending time with others, or spend most of their time alone, had higher levels of anxiety. This is consistent with previous literature where anxiety increased as a function of loneliness^80–82^ and higher anxiety was related to avoidant social behavior^83^. Consistent with the literature, anxiety scores were predominately higher in females, as compared to males^84–87^, and in young adults (aged 18–39 years), as compared to older adults aged (40–70)^87^. As others have published, anxiety scores were higher in participants indicating lower household income levels^88,89^ and lower education levels^90^. In alignment with other reports, anxiety scores also varied with different levels of employment where retired participants reported the lowest anxiety scores and participants that were unemployed^89,91^ or had more than one job had the highest levels of anxiety. Anxiety scores also varied with marital status, in alignment with other reports, where participants classifying as ‘single’, ‘separated’, and ‘living with partner’ reported higher anxiety than others (e.g., ‘married’, ‘divorced’, ‘widowed’)^89^. As reported elsewhere our participants reporting mixed race backgrounds had higher anxiety^92^ than other racial/ethnic groups (e.g., white, African American, Hispanic, Asian). Lastly, individuals who reported previous COVID-19 experienced more anxiety. This finding is consistent with other studies in adults^93–95^ and a longitudinal study of adolescents with anxiety disorders that found SARS-COV-2 infection was associated with a 30% worsening in anxiety severity^96^, regardless of treatment status. Altogether, this concordance with the literature supports the broader set of findings.

Most judgment variables showed significant differences between ‘higher’ and ‘lower’ anxiety groups, suggesting three general constructs. The first being that the ‘higher’ anxiety group had higher loss aversion^50,51^ which corresponds to an overweighting of bad outcomes relative to good ones^44^. The ‘higher’ anxiety group also had higher Peak Positive Risk and Total Reward Risk, indicating that there was a higher uncertainty that must be overcome to approach stimuli, and that the interactions between reward and the associated risk were higher. Both observations point to difficulties with initiating behavior toward positive things, per Markowitz’s decision utility equation^113^. The same participants had lower Peak Negative Risk as compared to the low anxiety group, indicating there was a lower uncertainty to avoid events. The ‘higher’ anxiety group also had lower Total Aversion Risk suggesting that interactions between aversion and the associated risk were lower. Similarly, they had lower Aversion Tipping Point which corresponds to the intensity of aversion beyond which avoidant choices are made, suggesting lower values in those with higher anxiety scores more readily make avoidant choices. Together, this set of judgment variables quantifies how individuals with high anxiety overweight bad outcomes relative to good ones, have difficulty approaching positive stimuli (i.e., more rewarding and non-aversive items), yet readily seem to avoid negative ones. A second construct suggests that individuals with difficulty approaching positive stimuli seem to be more open to risk-seeking. Participants with high anxiety scores had higher ante and lower risk aversion indicating they would be more willing to play a game with uncertain outcomes, and that they do not prefer actions that lead to certain outcomes (i.e., they prefer two birds in the bush vs. one in the hand). This contrasts with studies that used emotional and monetary stimuli to observe heightened risk aversion in individuals with anxiety^47,48^. This difference in observations might depend on how a question is placed in the context of gain or loss (i.e., framing effects)^44,97^. The third construct was identified by low loss resilience, tradeoff range, and consistency metrics. Specifically, a lower Tradeoff range in high-anxiety persons is consistent with a restrictive portfolio of positive and negative preferences. Lower Reward Aversion consistency suggests a likelihood of indifference or that a person neither likes nor dislikes a particular stimulus. People with high anxiety scores are less loss resilient, meaning they have a reduced ability to rebound from bad outcomes. The three constructs describe a behavioral profile for high anxiety persons as having less resilience, more avoidance, and more indifference behavior. Together, these three general groupings of judgment variables point to known features of anxiety^49^ but provide a lawful, quantitative framework^56,57^ for framing the condition and support the hypothesis that unique constellations of judgment variables underlie other mental health conditions like depression^65^ and suicidality^66^. The current findings support calls for the development of a standard model of mind^98^, albeit based on processes of judgment and agency as opposed to variables focused primarily on cognition.

Several limitations need be considered. First, the participants were recruited from the United States, and region and culture may influence the importance of judgment variables in predicting anxiety as psychiatric symptoms differ across cultures^99–104^. Second, participants with mental health conditions were oversampled to meet criteria for other survey components not discussed here. This oversampling could bias results and more generalized samples are needed to validate and extend our findings. Third, all variables were self-reported and not collected from clinical records or framed as a double-blinded trial with investigator-administered survey instruments. Fourth, the cohort was sampled during the COVID-19 pandemic, in which greater incidents of loneliness and anxiety have been reported^78,79^. It will be important to prospectively investigate if similar behavioral patterns predict anxiety in the absence of a pandemic. Fifth, the survey did not request participants to differentiate between white non-Hispanic and non-white; more in-depth questions regarding racial and ethnic backgrounds should be considered in future data collections.

The current study used a computational cognition framework to assess how biases in human judgment might contribute to predicting anxiety levels. Using a small set of judgment and contextual variables (including demographics, perceived loneliness, and COVID-19 history) with a balanced Random Forest framework, this study achieved high accuracies up to 88.51% and AUC ROC values of 0.69–0.74 for predicting state anxiety levels derived from the STAI^70^. Judgment variables were extracted from a short (2–3 min), simple, and unsupervised picture rating task that can be easily completed on a personal electronic device. In these prediction analyses, the four most important variables (age, employment, income, and loneliness) were contextual variables that contributed 29–33% of the relative importance and judgment variables contributed up to 61% of the relative importance for prediction. Furthermore, age, loneliness, and employment status significantly mediated and moderated the relationship between judgment variables and anxiety scores—indicating statistically mechanistic relationships between these variables, and suggesting that both cognitive variables and contextual variables are important for accurately predicting anxiety levels. Judgment variables differed across participants with higher and lower anxiety scores providing a behavioral profile for participants with higher anxiety scores. That is to say, individuals with higher anxiety scores overweighted bad outcomes relative to good ones, had difficulty approaching positive stimuli, yet readily avoided negative ones. Along with this higher avoidance, they also had lower resilience and higher indifference, consistent with prior reports^49^. This study supports the hypothesis that a small set of interpretable judgment and contextual variables can accurately predict psychiatric symptoms and provide a computational cognitive framework to better understand and classify anxiety and other mental health conditions.

## Methods

### Participant recruitment

Gold Research Inc. (San Antonio, Texas) recruited study participants from multiple vendors in December 2021. 4019 de-identified participants (mean age ± std = 51.4 ± 14.9 years) were randomly sampled from the general U.S. population using an email survey database accessed by Gold Research, Inc. and a double opt-in methodology as described in detail in refs. ^66,67,94,105,106^, and in the Supplemental Material. All participants provided informed consent following oversight by Northwestern University’s and the University of Cincinnati’s Institutional Review Board and in accordance with the Declaration of Helsinki (see “Ethical statement” and refs. ^66,67,94,105,106^). Participants were balanced to meet the U.S. Census Bureau’s demographic criteria at the time of the survey (December 2021) and oversampled by 15% of the sample for mental health conditions (see Supplemental Material). The survey was composed of several blocks of questions using questionnaires (detailed below) for depression, anxiety, suicidality, addiction, psychosis, violent ideation, disruptive and destructive behaviors, perceived loneliness, along with demographic, self-reported mental health, and COVID-19 history questionnaires. Participants also completed a 48-item picture rating task (Fig. 1) split into two 24 picture blocks.

### Ethical statement

Participation was offered with language noting that Gold Research was administering an emotional health questionnaire on behalf of Northwestern University, with the phrasing: “*We will be evaluating how different emotions and experiences are connected and may relate to our emotional health*.” All participants provided written informed consent, including their primary participation in the study and the secondary use of their anonymized, de-identified (i.e., all identifying information removed by Gold Research Inc. prior to retrieval by the research group) data in secondary analyses (see Supplemental Material). The study was approved by the Institutional Review Boards for Northwestern University (NU) and University of Cincinnati (UC) in accordance with the Declaration of Helsinki (approval number STU00213665 for NU and 2023-0164 for UC).

### Data filtering

Gold Research excluded participants using four criteria: (1) participants selected the same response throughout any section of the questionnaire (e.g., selecting option “1” for all questions), (2) participants indicated they had ten or more clinician-diagnosed illnesses out of a possible 17 (data not described here), (3) if both education level and years of education did not match, and (4) if they completed the questionnaire in less than 800 s. After filtering for these criteria, Gold Research provided the research team data from 4019 participants. These data were further screened using responses from the picture rating task. These procedures have been adapted from Azcona et al.^64^ and are detailed in the Supplemental Material under *Data filtering based on picture rating task*. In short, participants were excluded if there was minimal variance in picture ratings (i.e., all pictures were rated the same or varied only by one point) and the quantitative feature set derived from the picture rating task was incomplete and/or there were extreme outliers (see *Judgment variables from picture rating task* and Supplemental Material). Using these exclusion criteria, 3476 participants were cleared for statistical analyses.

### Contextual variables from survey questionnaires

Participants completed the survey using the online platform provided by Gold Research, Inc. Participants were asked to self-report (a) perceived loneliness in the past month (loneliness), (b) demographics including age, gender assigned at birth (sex), annual household income (income), marital status (marital), employment status (employment), level of education (edu), number of years of education (edu_years), race/ethnicity (race/ethnicity), and (c) two COVID-19 questions: (i) if the participant had ever tested positive for COVID-19 (test) and (ii) if the participant was ever diagnosed by a clinician with COVID-19 (diagnosis). The complete text regarding these questions is listed under the *Survey Questions* section in Supplemental Material. The response set to (a)–(c) is referred to as ‘contextual variables’ hereafter. Following data filtering as described above and in refs. ^94,105,106^, the 3476 participants were categorized as predominately female (61.5%), married (51.4%), white (85.7%), employed full-time (35.8%) with some college education (29.6%), and on average older (mean age = 51 years), see Supplementary Table 1 for a complete summary.

### Anxiety questionnaire

This study assessed anxiety in relation to contextual and picture rating-derived variables (described below). We used the State-Trait Anxiety Inventory (STAI) questionnaire which is commonly used to measure trait and state anxiety^70^. It is used in clinical settings to quantify anxiety. The STAI consists of 20 questions for current state anxiety, and 20 questions for trait anxiety. In this study, only the 20-state anxiety (STAI-S) questions were deployed in the online survey. Participants were instructed to answer each question based on a 4-point Likert scale (1 = Not at all; 2 = Somewhat; 3 = Moderately so; 4 = Very much so) based on how they feel *right now*, that is, at the time of the survey. The questions were scored following the instructions in the score key for the form Y-1 of STAI (https://oml.eular.org/sysModules/obxOml/docs/ID_150/State-Trait-Anxiety-Inventory.pdf). The scored sum of STAI-S ranged from 20 to 80 and is hereafter referred to as ‘STAI-S score’ and/or ‘anxiety score’. STAI-S score distributions are shown in Fig. 3 with three red arrows marking the threshold values used in classification as described under ‘Classification analysis’. The STAI thresholds of 35, 45, and 55 roughly corresponds to 50th percentile (median), 75th percentile and 90th percentile, respectively, of the STAI-S scores (Fig. 3).

### Picture rating task

Participants were shown 48 unique color images from the International Affective Picture System (IAPS)^68,69^. Six picture categories were used: (1) sports, (2) disasters, (3) cute animals, (4) aggressive animals, (5) nature (beach vs. mountains), and (6) adults in bathing suits, with eight pictures per category (48 pictures in total, a sample image is shown in Fig. 1A), with all pictures in a category having similar published calibration. These images act as mildly emotional stimuli that are employed to assess both positive and negative value (i.e., reward or liking vs. aversion or disliking) and have been broadly used and validated in research of human emotion, attention, and preference^68,69^. Images were displayed on participants’ personal devices with a maximum size of 1204 × 768 pixels. Below each picture was a rating scale from −3 (dislike very much) to +3 (like very much), where 0 indicated a neutral point (Fig. 1A). While there was no time limit for selecting a picture rating, participants were asked in the instructions to rate the images as quickly as possible and to use their first impression; specific instructions can be found in the Supplemental Material. Once a rating was selected, the next image was displayed.

### Judgment variables derived from a picture rating task

Data from the picture rating task were analyzed using a computational framework to characterize preference judgments. Referred to as relative preference theory (RPT)^56,57,62^, this framework has been adapted to derive judgment features from picture ratings^64,66,67^ as opposed to operant keypressing^52,56,57,59–62,107^. For each participant, picture ratings from each of the six image categories were split into two sets—positive and negative. For each of these two sets, and for all six categories, the mean, Shannon entropy^56,108^, and variance were calculated, yielding a tuple denoted as $\left({{\boldsymbol{K}}}^{{\boldsymbol{+}}}{\boldsymbol{,}}{{\boldsymbol{\sigma }}}^{{\boldsymbol{+}}}{\boldsymbol{,}}{{\boldsymbol{H}}}^{{\boldsymbol{+}}}\right)$ for the positive ratings and $\left({{\boldsymbol{K}}}^{{\boldsymbol{-}}}{\boldsymbol{,}}{{\boldsymbol{\sigma }}}^{{\boldsymbol{-}}}{\boldsymbol{,}}{{\boldsymbol{H}}}^{{\boldsymbol{-}}}\right)$ for the negative ratings. This resulted in a total of 36 $\left({\boldsymbol{K}}{\boldsymbol{,}}{\boldsymbol{\sigma }}{\boldsymbol{,}}{\boldsymbol{H}}\right)$ variables. Next, for each participant, the mean across the six categories was computed for each $\left({\boldsymbol{K}}{\boldsymbol{,}}{\boldsymbol{\sigma }}{\boldsymbol{,}}{\boldsymbol{H}}\right)$ variable, resulting in six additional variables: mean $\left({{\boldsymbol{K}}}^{{\boldsymbol{+}}}{\boldsymbol{,}}{{\boldsymbol{\sigma }}}^{{\boldsymbol{+}}}{\boldsymbol{,}}{{\boldsymbol{H}}}^{{\boldsymbol{+}}}\right)$ representing reward behavior and mean $\left({{\boldsymbol{K}}}^{{\boldsymbol{-}}}{\boldsymbol{,}}{{\boldsymbol{\sigma }}}^{{\boldsymbol{-}}}{\boldsymbol{,}}{{\boldsymbol{H}}}^{{\boldsymbol{-}}}\right)$ representing aversion behavior. For each participant, three separate curves for value, limit, and tradeoff functions (see Fig. 1B–D were plotted using MATLAB and the library *polyfit*, following other publications (see details in Supplemental Material)^56,57,62,64,107^. Representative curves from 500 randomly selected participants out of the 3476 cohort are shown in Supplementary Fig. 1.

Goodness of fit for these functions was assessed by computing ${R}^{2}$ values, adjusted ${R}^{2}$ values (accounting for degrees of freedom), and $F$-statistics for each participant’s model fit (Supplementary Table 2A). Individual participants’ $\left({\boldsymbol{K}}{\boldsymbol{,}}{\boldsymbol{H}}\right)$ value functions were fit by concave logarithmic, or power-law functions (Supplementary Table 2, Supplementary Fig. 1). ${R}^{2}$ values ranged from 0.85 to 0.94 for logarithmic fits of the value function, which was considered very high. Concave quadratic fits across individual participants’ $\left({\boldsymbol{K}}{\boldsymbol{,}}{\boldsymbol{\sigma }}\right)$ data are displayed in Supplementary Fig. 1, and goodness of fit assessed using the same metrics as with the $\left({\boldsymbol{K}}{\boldsymbol{,}}{\boldsymbol{H}}\right)$ data (Supplementary Table 2A). All ${R}^{2}$ values for the quadratic fits exceeded 0.80 and ranged from 0.84 to 0.96. Lastly, radial functions were fit to test for trade-offs in the distribution of ${{\boldsymbol{H}}}^{{\boldsymbol{-}}}$ and ${{\boldsymbol{H}}}^{{\boldsymbol{+}}}$ values across categories for each individual participant. Supplementary Fig. 1 displays radial fits across individual participants’ $\left({{\boldsymbol{H}}}^{{\boldsymbol{+}}}{\boldsymbol{,}}{{\boldsymbol{H}}}^{{\boldsymbol{-}}}\right)$ and $\left({{\boldsymbol{H}}}^{{\boldsymbol{+}}}{\boldsymbol{,}}{{\boldsymbol{H}}}^{{\boldsymbol{-}}}\right)$ data points for a random sample of participants.

The RPT framework fits reward/aversion curves and derives mathematical features from these graphical plots that are psychologically interpretable, scalable, recurrent, and discrete^56,57,64^. At least 15 variables can be extracted from this framework^64^, including Loss Aversion (LA)^45^, Risk Aversion (RA)^46^, Loss Resilience (LR), Ante, Insurance, Total Reward Risk (Total RR), Total Aversion Risk (Total AR), Peak Positive Risk (Peak PR), Peak Negative Risk (Peak NR), Reward Tipping Point (Reward TP), Aversion Tipping Point (Aversion TP), Reward-Aversion tradeoff (RA tradeoff), Tradeoff range, Reward-Aversion consistency (RA consistency) and Consistency range (Fig. 1B–D, Table 1). Each variable describes a quantitative component of the reward/aversion processing involved with judgment behavior (see details about each feature in Supplemental Material). The term ‘judgment variables’ will be used hereafter in reference to these features. Summary statistics for all 15 judgment variables obtained from all participants are summarized in Supplementary Table 2B.

### Classification analysis

All analyses were performed in R. Judgment variables and contextual variables, including demographics, perceived loneliness, and COVID-19 questions, were used in the classification analyses. Random Forest (*RF*) and balanced Random Forest (*bRF*) analyses were used to classify anxiety scores into ‘higher’ and ‘lower’ classes (see below). The open access package ‘randomForest’ in R was used to train the *RF* and *bRF* models on training dataset.

### Random Forest (RF) and balanced Random Forest (bRF) analysis

Anxiety scores were divided into two classes ‘higher’ and ‘lower’ classes based on three threshold values of 35, 45 and 55 as shown in Fig. 3 marked with red arrows. All values below a given threshold were labeled as ‘lower’ and values above and equal to the threshold were labeled as ‘higher’. Data were divided into train and test sets with a 70:30% ratio. *RF* and *bRF* approaches were implemented for each of the three thresholds using the command ‘randomForest’ from the package ‘randomForest’ in R. The number of variables randomly sampled as candidates at each split was 5 and the number of trees grown was 1000. The *bRF* performs random down-sampling of the majority class at each bootstrap sample to match the number of samples in majority and minority classes. The *bRF* approach was used in addition to the standard *RF* analysis because of the greater class imbalance when the anxiety score threshold was set to 45 and 55 (i.e., the ‘lower’ class occupied 70% and 88% of the dataset respectively). Note that *bRF* only performs down-sampling during training of the model. Once the model is trained, it calculates the prediction metrics on the complete, imbalanced train and test sets.

Out of bag (OOB) accuracy was reported for the training set. The model was then tested with the imbalanced test dataset and accuracy, sensitivity, specificity, AUC ROC (area under the receiving operating characteristics curve), and Balanced Accuracy (mean of sensitivity and specificity) were reported. Here, ‘higher’ was considered the positive class. For each threshold, percentages of each class relative to the entire dataset were reported. The entire procedure was repeated for each threshold value (i.e., 35, 45, 55). The multi-dimensional scaling (MDS) scaling coordinates of the proximity matrix for *RF* and *bRF* analyses were plotted to see how segregated the two clusters of lower and higher anxiety scores were, using the command ‘MDSplot’ in the package ‘randomForest’. The proximity matrix contains the frequency for each pair of data points. If two data points occupy the same terminal node through one decision tree, their proximity is increased by one. At the end of the run of all decision trees, the proximities are normalized by dividing by the number of trees. The MDS plots display how segregated the clusters were for the classification performed by *RF* or *bRF*.

To compare the performance of the classifiers to chance levels, permutation analysis was conducted for *RF* and *bRF* for each of the three threshold levels with 100 iterations. For each iteration, the ‘lower’/‘higher’ labels were shuffled randomly and *RF* and *bRF* analyses were run with the procedure described above. The above-mentioned output metrics were averaged over the 100 iterations to produce a model of chance effects.

### Relative importance of features

The judgment and contextual variables were sorted based on the mean decrease in Gini scores and were plotted by decreasing feature importance, with the most important features appearing on the top of the plot. Gini score is a fundamental outcome of the random forest algorithms as it shows for each feature how large was the discriminative value for separating the data points into different classes. That is, how important was each variable in the classification, or how much was the uncertainty reduced in the model, leading to accurate predictions. The higher the mean decrease Gini score, the more important the feature was for the classification. The relative importance of the features was analyzed by normalizing the Gini score of each feature to the sum of all Gini scores. This gives the relative proportion of importance for each feature, with the sum for all features being 1. The Gini score plots, and the relative importance of the features were used in this study as an associated sensitivity analysis for the random forest algorithms.

### Post hoc analysis: mediation and moderation analysis

Mediation and moderation were used as post hoc analyses to understand how judgment variables and the most important contextual variables, based on Gini scores, interact to model anxiety scores. These statistical mechanisms aide interpretation of the prediction results and follow procedures we have published before^109,110^.

### Mediation analysis

Mediation was utilized to elucidate statistically causal relationship between judgment variables, contextual variables, and anxiety scores. The mediation model defines the relationship between an independent variable (X) and dependent variable (Y) with the inclusion of a third mediator variable (Me). Two sets of analyses were conducted where Y was the anxiety score and: (i) X were each of the judgment variables and Me were each of the most important contextual variables based on Gini score from *RF* and *bRF* analyses (i.e., the top four scores); (ii) X were the most important contextual variables and Me were the judgment variables.

The mediation model proposes that instead of a direct statistical causal relationship between X and Y, X influences Me, which then influences Y. Beta coefficients and their standard error (s) terms from the following linear regression equations, following the four-step process of Baron and Kenny (1986)^111,112^, were used to calculate Sobel *p*-values and mediation effect percentages (*T*_*eff*_):

*Step 1-3*:

1 $$Y={\gamma }_{1}+c\left(X\right)+{\epsilon }_{1}$$

2 $${Me}={\gamma }_{2}+a\left(X\right)+{\epsilon }_{2}$$

3 $$Y={\gamma }_{3}+{c}^{{\prime} }\left(X\right)+b\left({Me}\right)+{\epsilon }_{3}$$

*Step 4*: Sobel’s test was then used to test if ${c}^{{\prime} }$ was significantly lower than $c$ using the following equation:

4 $${{Sobel}\,{z}}-{score}=\frac{c-{c}^{{\prime} }}{\sqrt{{b}^{2}{s}_{b}^{2}+{a}^{2}{s}_{a}^{2}}}=\frac{{ab}}{\sqrt{{b}^{2}{s}_{b}^{2}+{a}^{2}{s}_{a}^{2}}}$$

Using a standard 2-tail z-score table, the Sobel *p*-value ($${p}_{{Sobel}})$$ was determined from the Sobel z-score and the mediation effect percentage (*T*_*eff*_) was calculated using the following equation:

5 $${T}_{{eff}}=100* \left[1-\frac{{c}^{{\prime} }}{c}\right]$$

Mediation was considered significant if *p*-values associated with terms *a*, *b*, and *c* were <0.05 from *Step 1–3* and ${p}_{{Sobel}}$ < 0.05^111^ and ${T}_{{eff}}$ > 50%^109,110^.

Secondary mediation analysis was run by switching variables assigned to X and Me to see if the mediation effects were directed. If ${p}_{{Sobel}}$ > 0.05 and ${T}_{{eff}}$ < 50% for the secondary mediation analysis, this supported that Me was in the causal pathway between X and Y.

### Moderation analysis

The moderation model proposes that the strength and direction of the relationship between an independent variable (X) and dependent variable (Y) is controlled by the moderator variable (Mo). In this study, X were each of the judgment variables, Mo were each of the most important contextual variables based on Gini score from *RF* and *bRF* analysis, and Y was the anxiety score. Moderation is characterized by the interaction term between X and Mo in the linear regression equation as given below:

6 $$Y={\beta }_{0}+{\beta }_{1}X+{\beta }_{2}{Mo}+{\beta }_{3}\left(X* {Mo}\right)+\epsilon$$

Moderation was considered significant if ${p}_{{\beta }_{3}}\le 0.05$ (the interaction term ${\beta }_{3}$ is significantly different than zero) and ${p}_{{overall}}\le 0.05$ (for the overall model)^109,110^. To check if the overall model was significant, we used F-test.

To test if the coefficient of the interaction term (${\beta }_{3})$ was significantly different than zero, we built full and restricted models and used partial F-tests to test the null hypothesis.

Full model:

7 $${\rm{Y}}={\beta }_{0}+{\beta }_{1}X+{\beta }_{2}{Mo}+{\beta }_{3}\left(X* {Mo}\right)+\epsilon$$

Restricted Model:

8 $$Y={\beta }_{0}+{\beta }_{1}X+{\beta }_{2}{Mo}+\epsilon$$

Null hypothesis:

9 $${H}_{0}:{\beta }_{3}=0$$

Alternative hypothesis:

10 $${H}_{A}:{\beta }_{3}\ne 0$$

If ${p}_{{\beta }_{3}}$, associated with the partial F-test was less than 0.05, we rejected our null hypothesis regarding the interaction term.

### Post hoc analysis: variable differences by anxiety score

These post hoc analyses assessed if the contextual variables and judgment variables differed by anxiety score.

### Contextual variable differences

Anxiety scores were assessed for differences by the different levels of contextual variables (except years of education) using Wilcoxon rank-sum test for questions with two levels and Kruskal Wallis test for questions with more than two levels. The ten contextual variables tested were loneliness, age, sex, income, marital status, employment status, education level, ethnicity, and COVID-19 test and diagnosis. Boxplots and *p*-values were reported.

### Judgment variable differences

Since judgment variables were continuous, they were divided into corresponding ‘higher’ and ‘lower’ groups following the respective grouping of anxiety scores at each of the three thresholds (35, 45, and 55), and tested using the one-sided Wilcoxon rank-sum test. Alternative hypotheses for each test, and the respective *p*-values, were reported. Bonferroni correction was done across all six tests (two tests for each of the three thresholds) for each judgment variable. The alternative hypothesis indicated if the judgment variable distributions differed between participants in ‘higher’ and ‘lower’ anxiety classes (for example, if a given judgment variable was higher in the ‘higher’ anxiety class as compared to the ‘lower’ anxiety class, or vice versa).

### Supplementary information

Supplemental Material

Supplemental Appendix 1

Supplemental Appendix 2

## Data Availability

Data were de-identified before being provided to the investigators. Data are available in Microsoft Excel format and include relative preference variables, demographic metrics and survey variables inclusive of anxiety variables. The data may be accessed in Appendix 1, Supplementary Information.

## Code availability

Computational behavior analysis used code published in refs. Azcona et al. 2022 and Kim et al. 2010. ML analyses used parameters as detailed in the Methods and Appendix 2, Supplementary Information. Mediation/moderation analyses used code sequences as detailed in refs. Bari et al. 2021 and Vike et al. 2022.
