# Supplementary material for: A novel approach to anxiety level prediction using small sets of judgment and survey variables
Source: Npj Ment Health Res. 2024 Jun 18;3:29. doi: 10.1038/s44184-024-00074-x (PMC11189415; doi:10.1038/s44184-024-00074-x)
Supplement: Supplementary file 1 — Supplemental Material [file 44184_2024_74_MOESM1_ESM.pdf]

Supplementary Methods

Participant Recruitment

Questionnaire responses were collected in December 2021. Participants between 18-70 years of age were recruited by Gold Research Inc. (San Antonio, Texas) using multiple methods: (1) by invitation only using customer databases from large companies that participate in revenue-sharing agreements, (2) via social media, (3) through direct mail, or (4) by volunteering to participate in research studies in lieu of product-based incentives such as coupons for everyday household purchases *(1–3)*. All participants were reimbursed $10 for their participation. Recruited respondents followed a double opt-in consent procedure to participate in the study (see below in **Ethical Statement**); during this process, they also provided demographic attributes including age, race, gender and others. This information was used to ensure that recruited participants represented the U.S. Census at the time of the survey (December 2021). During the study, respondents were also prompted with repeated test questions to screen out those providing random and illogical responses and/or those showing flatline or speeder behavior. Data from those flagged as non-adherers were removed. To ensure adequate samples of participants with mental health conditions, Gold Research oversampled 15% (60,000/400,000) of the sample for mental health conditions. Gold Research reported that >400,000 respondents were contacted to complete the questionnaire. Gold Research estimated that of the >400,000 participants, >300,000 (75%) either did not respond or declined participation. Of the remaining 25% (100,000/400,000) who clicked on the survey link, >50% did not fully complete the questionnaire. Of the ≥48,000 participants who completed the survey, those who did not clear data integrity assessments were omitted. Participants meeting quality assurance procedures (including survey completion as described in **General Data Exclusion**) were selected, with a limit of 4000-4050 total participants. Eligible participants were required to be between 18-70 years of age at the time of the questionnaire, could comprehend the English language, and had access to an electronic device (e.g., laptop, cell phone). Data from 4019 participants (mean age ± std = 51.4 ± 14.9 years) passed Gold Research's initial data integrity assessments, were anonymized, and then sent to the research team. Participants provided informed consent as described in **Ethical Statement**.

Survey Questions

For initial recruitment, participants received the following communication:

Gold Research Inc., a national market research firm and its client, Northwestern University, request your participation in this study of emotional health. We will be evaluating how different emotions and experiences are connected and may relate to our emotional health. The information you provide will be kept confidential, coded to be anonymous so it cannot be connected back to you and will be used only for research purposes. Researchers will not be able to contact you or restudy you after this survey. We will not share your information with any other third party. We will also not use your information to identify you individually or use your responses to market or sell other services or products to you. As part of this effort, you will not be asked to provide any personal identifiers such as your name, email, phone number, address or social media handles. A unique identifier will be generated for you and each survey participant to enhance privacy. As part of the survey process, we will be able to tell if you completed the survey, but we will not be able to tell which answers were yours. For this study, we are going to ask you some questions about yourself and how much you like or dislike a set of pictures. You may discontinue this study at any time. We appreciate your help with this study, given the serious challenges facing many people regarding emotional health at this time. We thank you in advance.

1. *Accept*
2. *Decline*

If participants responded with "Accept", they were sent a further communication with the following:

Thank you for participating in our survey. All responses during this survey are anonymous and confidential. We will be able to tell if you completed the survey, but we will not be able to tell which answers were yours. In this study, we aim to understand how different emotions and experiences relate to visual processing.

We are going to:

****Ask you some questions about yourself***

****Have you rate how much you like or dislike a set of pictures***

For this study, your identity is protected and your answers are anonymous and confidential. Press "Next" to proceed.

The survey would then begin if participants pressed “Next”.

Out of the larger survey, the questions regarding self-reported perceived loneliness, demographics, COVID-19 history are listed below using the abbreviations described in the main text for this study. Given the state part of State-Trait Anxiety Inventory (STAI) is a published and validated instrument *(4)*, it is not detailed below.

<u>Loneliness:</u>

Please rate how often you have experienced each of the following on a scale of 1-5.

You avoid spending time with others or you spend most of your time alone (in the past one month):

1) Never 2) Rarely 3) Sometimes 4) Often 5) Always

<u>Age:</u>

Please indicate your age (scroll bar response with integer years).

<u>Sex:</u>

Are you…(gender assigned at birth):

1. Male
2. Female
3. Other or Prefer Not to Answer

<u>Income:</u>

Which of the following best reflects your annual household income?

1. Under $25,000
2. $25,000 but less than $50,000
3. $50,000 but less than $75,000
4. $75,000 but less than $100,000
5. $100,000 but less than $150,000
6. $150,000 but less than $300,000

7. $300,000 or more

Marital:

Which of the following best describes your marital status?

1. Single
2. Married
3. Divorced
4. Separated
5. Widowed
6. Living with partner
7. I prefer not to answer this question

Employment:

Please describe your current employment situation…?

1. Unemployed
2. Full-time employment
3. Part-time employment
4. Self-employed
5. More than one job
6. Retired
7. Other

There are two pieces of information asked for in the following question.

Select the highest level of education you have achieved, and

Enter the total number of the years you completed below.

Edu:

Please select the highest level of education you have achieved:

1. Some High School — Enter a number between 8-12 below
2. High School Graduate — Enter a number between 11-14 below
3. Some College — Enter a number between 13-16 below

4. Bachelor Degree — Enter a number between 15-17 below
5. Some Graduate School — Enter a number between 15-21 below
6. Graduate Degree — Enter a number 18 or greater below
7. Post-Doctoral Training — Enter a number greater than 18 below

<u>Edu_years:</u>

Please indicate the total number of years of education you completed.

<u>Race:</u>

Do you consider yourself...(select only one response)

1. White
2. African American
3. Hispanic
4. Asian or Pacific Islander
5. Native American or Alaskan Native
6. Mixed Racial Background
7. Other Race
8. Prefer Not To Answer

<u>Test:</u>

Have you ever tested positive for COVID-19? Y/N

<u>Diagnosis:</u>

Have you ever been diagnosed with COVID-19 by a medical clinician? Y/N

Picture Rating Task

The instructions displayed before the start of the picture rating task in the survey were as follows:

"The next part of this survey involves looking at pictures and then responding how much you like or dislike the image. Please rate each image on a scale from -3 (Dislike Very Much) to +3 (Like Very Much). Zero (0) is neutral... meaning you have no feelings either way. The images are a set of photographs that have been used by scientists around the world for over 20 years. It is important

you rate each picture based on your initial emotional response. There are no right or wrong answers... just respond with your feelings, and rate the pictures very quickly. Use the drop down box to select rating between -3 and 3."

Participants were then shown two sets of 24 pictures with survey questions in between the two sets.

General Data Exclusion

A general set of quality assurance procedures were implemented as first used in other publications *(1–3)*. Following this framework, participants meeting at least one of six criteria were removed from the cohort: (1) participants whose relative preference analysis yielded extreme outliers > 3 IQRS or incomplete measurements and met three other criteria for relative preference computation (please see next section), (2) participants that had mis-matching responses to years of education and education level in the survey, (3) participants with ten or more clinician-diagnosed illnesses, (4) participants that selected the same response for at least one section of the survey, (5) participants that rated all images in the behavioral task the same or with a variance of 1 (meaning only two of 7 Likert points were used), (6) participants that completed the questionnaire in less than 800 seconds *(1–3)*.

Judgment variables from picture rating task

This study used a relative preference theory (RPT) feature space for characterizing preference judgments in individuals *(5–7)*, which has been tightly linked to reward/aversion circuitry in the human brain *(8–13)*.

For each participant, ratings of images in each of the six image categories were split into two sets – positive and negative ratings. These positive and negative ratings for all six categories were then used to compute the mean, Shannon entropy and variance, yielding a tuple denoted as $(\boldsymbol{K}^+, \boldsymbol{\sigma}^+, \boldsymbol{H}^+)$ for the positive ratings and $(\boldsymbol{K}^-, \boldsymbol{\sigma}^-, \boldsymbol{H}^-)$for the negative ratings within each picture category. It should be noted that for computation of ***H***, data was screened for cases where ***K***=0 for a given category (i.e., cases where the subject made all neutral ratings to neither approach nor to avoid any picture in a given category). Computation of ***H*** for a given picture category requires that be ***K***>0 given that *H* computation results in an undefinable $\log_{10}(0/0)$ when ***K***=0. In such cases, ***H*** was set to 0 for categories in which the subject rated "0" for all the stimuli. These computations

resulted in 12 $(\boldsymbol{K}, \boldsymbol{\sigma}, \boldsymbol{H})$ tuples, or a total of 36 variables. For each participant, the mean across six categories was also computed for both positive and negative ratings which resulted in additional six variables: $(\boldsymbol{K}^+, \boldsymbol{\sigma}^+, \boldsymbol{H}^+)$ representing reward behavior, and $(\boldsymbol{K}^-, \boldsymbol{\sigma}^-, \boldsymbol{H}^-)$ representing aversion behavior.

For fitting models to participants' ratings, data was further screened for inclusion/exclusion criteria was as follows:

1. Valid entropy (***H***) calculations (as described above),
2. Exclusion of extreme outliers in the variables extracted from the graphs as follows – loss aversion values > 200 (resulting in N=42 exclusions) and positive quadratic area > 100 (resulting in N=5 exclusions).
3. At least three points for a non-linear fit, or sufficient data points to fit the model with a computable R^2, and
4. Consistency of individual and group models. This last criterion meant that the curve concavity for individual subject fits be consistent with the curve concavity of the group-level fits (and boundary envelopes).

Criteria (3) and (4) are important operational definitions since there is a potential for convergence failures with curve fitting. Overall, 3476 participants out of 4019 met all quality assurance criteria for picture rating data and survey data.

Next, for each participant, three separate curves for value function, limit function and tradeoff function (see **Figure 2(B-D**) were plotted with this data, detailed below, using MATLAB and the library *polyfit*. These curves from 500 randomly selected participants are shown in **Figure S1**. Subsequently, judgment variables were extracted from each of the participants' curves. The curves are briefly detailed below, followed by a description of the judgment variables extracted from each curve (for more details, see Azcona et al. 2022 *(14)*).

An array of judgment variables was extracted from each of the three curves (**Figure 2(B)-(D)** and **Table 1**). These variables are described below in psychological terms, and organized relative to the curve from which they were extracted:

(1) The (***K, H***) curve, which consists of ($\boldsymbol{K}^+, \boldsymbol{H}^+$) on the positive x-axis and ($\boldsymbol{K}^-, \boldsymbol{H}^-$) on the negative x-axis is analogous to the value function from Kahneman's Prospect Theory. The features that were extracted from this curve include: *Loss Aversion (LA), Risk Aversion (RA), Loss Resilience (LR), Ante and Insurance.*

Loss Aversion (LA): Loss aversion was computed as the absolute value of the ratio of the linear regression slope of (log $\boldsymbol{K}^-$, log $\boldsymbol{H}^-$) to the linear regression slope of (log $\boldsymbol{K}^+$, log $\boldsymbol{H}^+$). It intuitively measures the degree to which one overweighs losses relative to gains. It is a fundamental measure in Kahneman and Tversky's Prospect Theory.

Risk Aversion (RA): Risk aversion is extracted as the ratio of the second derivative of the ($\boldsymbol{K}^+$, $\boldsymbol{H}^+$) curve to its first derivative, which itself produces a curve. To produce a unitary value for prediction, we calculated Risk Aversion at $K^+ = 0.5$. Informally, RA measures the degree to which an individual prefers a likely reward in comparison to a better but more uncertain reward. RA is a common notion in economics that studies decision-making under uncertainty.

Loss Resilience (LR): Loss resilience is defined to be the absolute value of the ratio of the second derivative of the ($\boldsymbol{K}^-$, $\boldsymbol{H}^-$) curve to its first derivative, which also produces a curve. For prediction, we calculated LR at $K^- = 1.5$. Informally, loss resilience is the degree to which an individual prefers to lose a small defined amount in comparison to losing a greater amount with more uncertainty associated with this loss.

Ante: Ante is the value of $\boldsymbol{K}^+$ when setting $\boldsymbol{H}^+ = 0$. It intuitively measures the stake one needs to engage in a game of chance and models the amount of a bid an individual is willing to make to enter a game of chance like poker.

Insurance: Insurance is the value of $\boldsymbol{K}^-$ when setting $\boldsymbol{H}^- = 0$. It intuitively measures how much insurance an individual might need against bad outcomes. It mirrors the ante, but in the framework of potential losses.

(2) The ($\boldsymbol{K},\boldsymbol{\sigma}$) curve, which consists of ($\boldsymbol{K}^+$, $\boldsymbol{\sigma}^+$) on the positive x-axis and ($\boldsymbol{K}^-$, $\boldsymbol{\sigma}^-$) on the negative x-axis is analogous to the mean-variance curve derived from portfolio theory *(15)*. It calibrates preference magnitude (i.e., **K**) relative to the variance in rewards and sanctions. The ($\boldsymbol{K}$, $\boldsymbol{\sigma}$) curve models the following question: Would an individual prefer a dollar with probability one, or value drawn from a normal distribution with mean of two and variance of two? The features that are

extracted from this curve include: *Peak Positive and Negative Risk, Reward and Aversion Tipping Point, Total Reward and Aversion Risk.*

Peak Positive Risk (Peak PR): The peak positive risk is value of $\boldsymbol{\sigma}^+$ where the derivative d $\boldsymbol{\sigma}^+$/d $\boldsymbol{K}^+$ is equal to zero. Intuitively, this represents maximum variance for approach behavior. It models where increases in positive value transition from a relationship with increases in risk, to a relationship with decreases in risk. Markowitz described decision utility as dU = **K** - b$\boldsymbol{\sigma}$ *(15)*, so Peak PR models when variance changes from potentially weighting against a decision to facilitating a decision.

Peak Negative Risk (Peak NR): The peak negative risk is value of $\boldsymbol{\sigma}^-$ where the derivative d $\boldsymbol{\sigma}$-/d $\boldsymbol{K}^-$ is equal to zero. Intuitively, this represents the maximum variance for avoidance behavior. Like with the peak positive risk, this transition point is important to consider for avoidance decisions in the context of Markowitz's decision utility *(15)*.

Reward Tipping Point (Reward TP): The reward tipping point is the value of $\boldsymbol{K}^+$ where the derivative d $\boldsymbol{\sigma}^+$/d $\boldsymbol{K}^+$ is equal to zero. Intuitively, this represents the rating intensity with maximum variance for approach behavior, and signals the amount of reward needed before an individual potentially decides to approach a goal-object.

Aversion Tipping Point (Aversion TP): The aversion tipping point is the value of $\boldsymbol{K}^-$ where the derivative d$\boldsymbol{\sigma}^-$/d$\boldsymbol{K}^-$ is equal to zero. Intuitively, this represents the rating intensity with maximum variance for avoidance behavior, and signals the amount of aversive value needed before an individual potentially decides to avoid a goal-object.

Total Reward Risk (Total RR): The total reward risk is the area under the curve on the positive quadrant of the graph of ($\boldsymbol{K}^+$, $\boldsymbol{\sigma}^+$). Intuitively, this variable represents the relationship between $\boldsymbol{K}^+$ and $\boldsymbol{\sigma}^+$ and can be thought of as a quantity that measures the amount of value one overall associates to positive stimuli.

Total Aversion Risk (Total AR): The total aversion risk is the area under the curve on the negative quadrant of the graph of ($\boldsymbol{K^-}$, $\boldsymbol{\sigma^-}$). Intuitively, this variable represents the relationship between $\boldsymbol{K^-}$ and $\boldsymbol{\sigma^-}$ and can be thought of as a quantity that measures the amount of value one overall associates to negative stimulus.

(3) The ($\boldsymbol{H^+}$, $\boldsymbol{H^-}$) curve allows the comparison of patterns in approach and avoidance judgments. The RPT Features that are extracted from this curve include: *Reward-Aversion tradeoff, Tradeoff Range, Reward-Aversion Consistency and Consistency Range.*

Reward-Aversion tradeoff (RA tradeoff): The reward-aversion tradeoff is the mean of the polar angles of the points in the ($\boldsymbol{H^+}$, $\boldsymbol{H^-}$) plane. Intuitively, this measures the mean ratio of entropies or patterns in approach to avoidance behavior.

Tradeoff range: This is the standard deviation of the polar angles of the points in the ($\boldsymbol{H^+}$, $\boldsymbol{H^-}$) plane. Intuitively, this measures the standard deviation in the patterns of approach and avoidance behavior. This variance represents the spread for positive preferences and negative preferences across a set of potential goal-objects and is one measure of the breadth of an individual's (or group's) preferences.

Reward-Aversion consistency (RA consistency): The reward-aversion consistency measures the mean of the distances of the data points in the ($\boldsymbol{H^+}$, $\boldsymbol{H^-}$) curve to the origin. Intuitively, this defines how individuals can have strong preferences (i.e., biases) for the same thing, reflecting *conflict*, or having low preferences for something, reflecting *indifference*. This gets at the consistency or compatibility of approach and avoidance, and how you can both like and dislike something, or be indifferent to both its positive and negative features.

Consistency Range: The consistency range measures the standard deviation of the distances of the data points in the ($\boldsymbol{H^+}$, $\boldsymbol{H^-}$) plane to the origin. Intuitively, this measures how the points in the $\boldsymbol{H^+H^-}$ plane vary about radial distance from the origin. The variance in this radial distance will reflect how much an individual goes between having *conflicting* preferences and having *indifferent* ones.

Supplementary Table 1. Summary of contextual variables for 3476 participants. S.D. = standard deviation.

Demographic Variable	Mean (S.D.)	Range	
age (years)	51.40 (14.92)	18-70	
education (years)	15.44 (3.36)	8-25	
**Contextual Variable**	**Level Description**	**Count**	**Percentage**
loneliness	(1)Never	2143	61.65
	(2)Rarely	367	10.56
	(3)Sometimes	485	13.95
	(4)Often	324	9.32
	(5)Always	157	4.52
sex	Male	1324	38.09
	Female	2138	61.51
	Other/Prefer not to answer	14	0.40
income ($)	<25,000	626	18.01
	25-50,000	906	26.06
	50-75,000	694	19.97
	75-100,000	526	15.13
	100-150,000	445	12.80
	150-300,000	230	6.62
	>300,000	49	1.41
marital	Single	766	22.04
	Married	1788	51.44
	Divorced	416	11.97
	Separated	64	1.84
	Widow	152	4.37
	Live with partner	269	7.74
	Other/Prefer not to answer	21	0.60
employment	Unemployed	481	13.84
	Full time	1244	35.79
	Part time	312	8.98
	Self-employed	165	4.75
	More than one job	7	0.20
	Retired	1045	30.06
	Other/Prefer not to answer	188	5.41
education	Some high school	101	2.91
	High school graduate	734	21.12
	Some college	1030	29.63
	Bachelor degree	777	22.35
	Some graduate school	176	5.06
	Graduate school graduate	564	16.23
	Post graduate/doctorate	94	2.70
race	White	2980	85.73
	Black/African American	227	6.53
	Hispanic	123	3.54
	Asian/Pacific Islander	122	3.51
	Native American/Alaskan Native	26	0.75
	Mixed	30	0.86
	Other	16	0.46
	Prefer not to answer	24	0.69
test	Yes	422	12.14
	No	3054	87.86
diagnosis	Yes	328	9.44
	No	3148	90.56
TOTAL N		3476	100.00

Supplementary Table 2. Summary fits of judgment variables for 3476 participants
(A) Goodness of fit statistics are displayed for individual logarithmic fits (log *K*, *H*) and quadratic (K, σ) fits of the picture rating groups of the IAPS images. For both the positive (approach) and negative (avoidance) curves, goodness of fit statistics are assessed through coefficient of determination (R^2) values, coefficient of determination values adjusted for degrees of freedom (R_{adj}^2) and *F-test* values (*F-value)*. Mean and Standard Deviation (SD) of the goodness of fit statistics are reported. Participants with less than two of the six picture rating categories were not included in fits. Values are rounded off at two decimal places. **(B)** Fifteen judgment features, which are derived from the (*K*,*H*), (*K*, σ) and (H^+,H^-) curves of the IAPS picture rating groups. Five features are derived from the value function *(K*,*H*), six from the quadratic function, and four from the trade-off function (H^+,H^-). Mean, standard deviation (SD), standard error of mean (SEM), and 95% confidence intervals of the values of the fifteen judgment features are reported for the cohort. Means are rounded off at two decimal places. Standard deviations (SD) are rounded off at three decimal places. Standard errors of means (SEM) are rounded off at four decimal places. Finally, the 95% confidence interval is reported to two decimal places.

(A)

Curve Set	Curve	Statistic	Mean (SD)
$(\log K, H)$ **logarithmic fit**	(log K^+, H^+)	R^2	0.92 (0.13)
		adjusted R^2	0.87 (0.21)
		F-value	1.90×10^{30} (2.06×10^{31})
	(log K^-, H^-)	R^2	0.96(0.07)
		adjusted R^2	0.89 (0.29)
		F-value	3.61×10^{30} (2.34×10^{31})
(K, σ) **quadratic fit**	(K^+,σ^+)	R^2	0.90 (0.13)
		adjusted R^2	0.84 (0.21)
		F-value	1.12×10^{30} (1.5×10^{31})
	(K^-, σ^-)	R^2	0.94 (0.1)
		adjusted R^2	0.9 (0.17)
		F-value	2.26×10^{30} (5×10^{31})

(B)

RPT Feature	Statistic	Value
Risk aversion	Mean (SD)	0.34 (0.125)
	SEM	0.0022
	95% CI	[0.34, 0.35]
Ante	Mean (SD)	0.17 (0.106)
	SEM	0.0018
	95% CI	[0.17, 0.18]
Loss resilience	Mean (SD)	0.32 (0.134)
	SEM	0.0024
	95% CI	[0.32, 0.33]
Insurance	Mean (SD)	-0.21 (0.108)
	SEM	0.0018

	95% CI	[-0.21, -0.21]
Loss aversion	Mean (SD)	0.88 (0.365)
	SEM	0.0063
	95% CI	[0.86, 0.89]
Peak positive risk	Mean (SD)	1.33 (0.290)
	SEM	0.0049
	95% CI	[1.32, 1.34]
Reward tipping point	Mean (SD)	1.48 (0.195)
	SEM	0.0034
	95% CI	[1.47, 1.48]
Total reward risk	Mean (SD)	2.71 (0.759)
	SEM	0.0130
	95% CI	[2.68, 2.73]
Negative apex	Mean (SD)	1.52 (0.349)
	SEM	0.0059
	95% CI	[1.51, 1.53]
Aversion tipping point	Mean (SD)	1.48 (0.103)
	SEM	0.0019
	95% CI	[1.48, 1.49]
Total aversion risk	Mean (SD)	3.05 (0.815)
	SEM	0.0139
	95% CI	[3.02, 3.08]
Reward-aversion tradeoff	Mean (SD)	50.04 (11.499)
	SEM	0.1950
	95% CI	[49.66, 50.42]
Tradeoff range	Mean (SD)	40.73 (7.024)
	SEM	0.3203
	95% CI	[40.10, 41.36]
Reward-aversion consistency	Mean (SD)	2.52 (0.256)
	SEM	0.0115
	95% CI	[2.50, 2.55]
Consistency range	Mean (SD)	0.47 (0.298)
	SEM	0.0133
	95% CI	[0.44, 0.49]

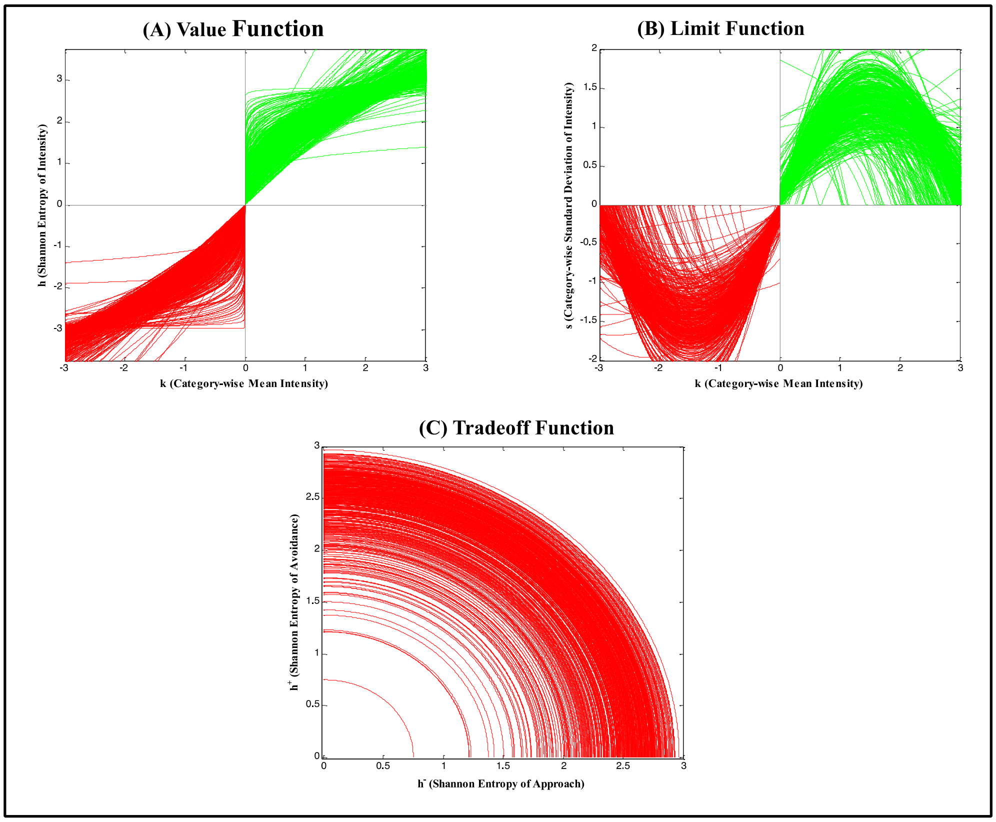

Supplementary Figure 1. Visual representation of the relative preference theory (RPT) **(A)** *value function*, **(B)** *limit function* and **(C)** *tradeoff function* curve fitting for 500 randomly selected participants out of 3476.

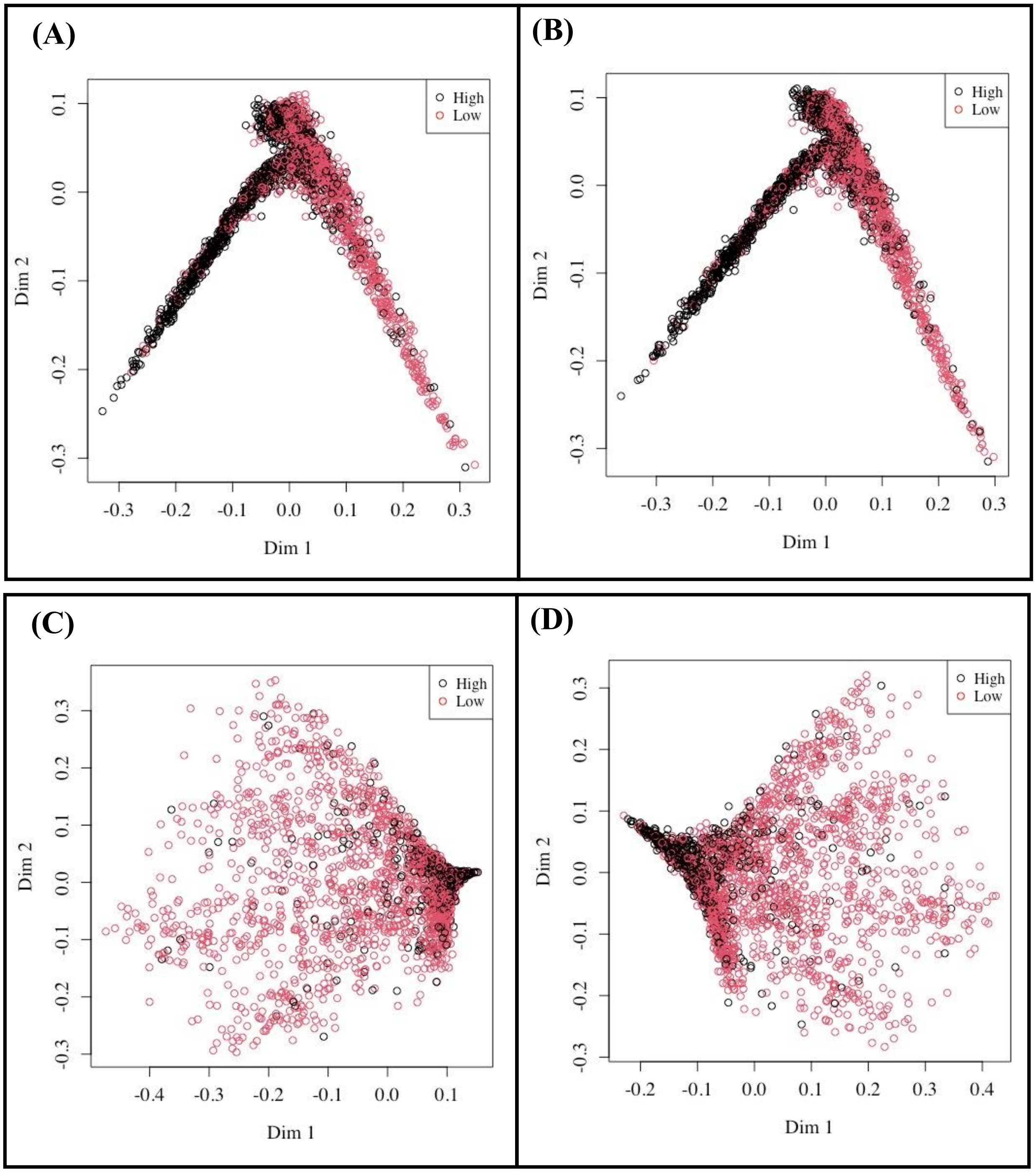
(A)
(B)
(C)
(D)
High
Low
Dim 1
Dim 2

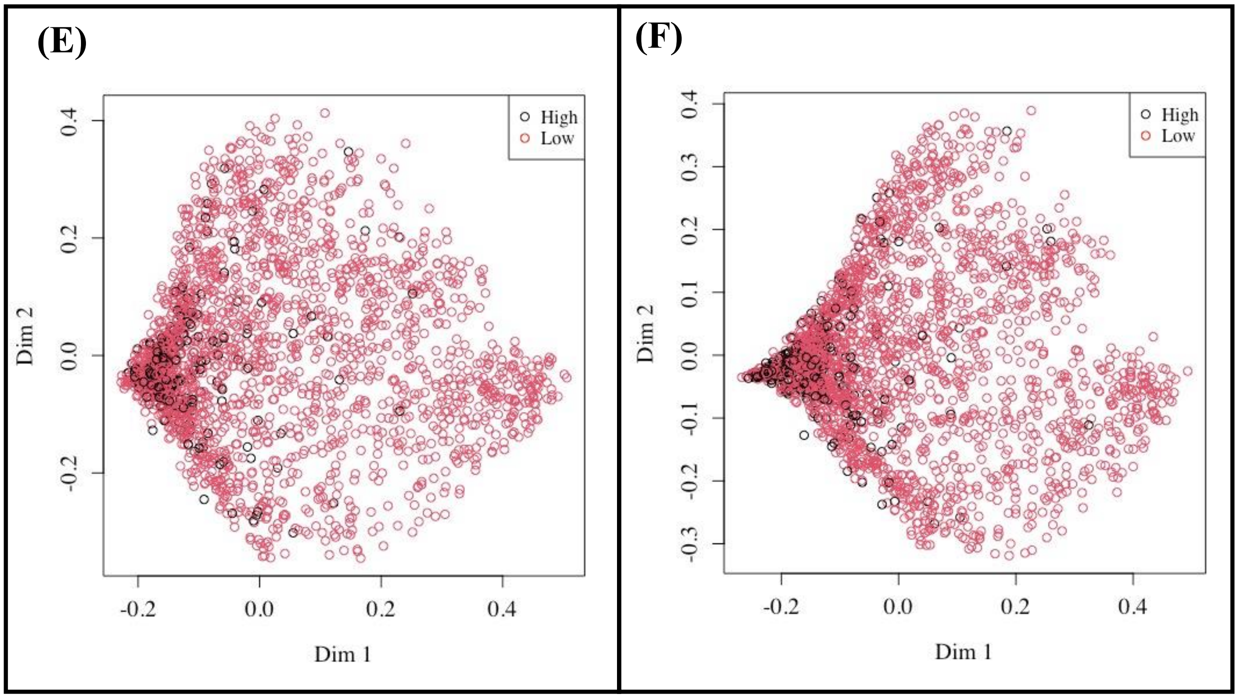

Supplementary Figure 2. Plots of multi-dimensional scaling (MDS) scaling coordinates of the proximity matrix for Random Forest (*RF*) and balanced Random (*bRF*) analyses, characterizing how fine the data segregation was by each analysis. Plots **(A)**, **(C)**, **(E)** corresponds to *RF* analyses with thresholds of 35, 45 and 55 respectively and **(B)**, **(D)**, **(F)** corresponds to *bRF* with thresholds of 35, 45 and 55 respectively.

Supplementary Table 3. Results of permutation analyses with 100 iterations for *Random Forest (RF)* and *balanced Random Forest (bRF)* for prediction of anxiety/STAI-S levels. For each iteration, 'lower (L)'/'higher (H)' labels were shuffled randomly and the classifer was run. The output metrics were averaged over 100 iterations. OOB =out of bag; AUC ROC = area under the receiving operating characteristics curve.

STAI-S Threshold	Percentage of data		Classifier	OOB Accuracy	Accuracy	Sensitivity	Specificity	ROC AUC	Balanced Accuracy
	Class 'H'	Class 'L'							
35	50.09	49.91	RF	50.10	49.93	50.96	49.06	0.4999	50.01
			bRF	50.10	49.93	50.19	49.75	0.4996	49.97
45	30.44	69.56	RF	68.97	69.22	1.10	98.84	0.4997	49.97
			bRF	62.59	62.65	18.18	82.00	0.5008	50.09
55	12.02	87.97	RF	87.99	87.83	0.06	99.96	0.5001	50.01
			bRF	80.98	80.68	9.15	90.53	0.4986	49.84

Supplementary Table 4. Detailed mediation results. Mediation was considered if p_a, p_b, p_c < 0.05 from *Step 1-3* and p_{Sobel} < 0.05 and T_{eff} > 50%. For primary mediation analysis X were judgment variables, Me were the four most important contextual variables (from **Figure 3** and **Table 3**) and Y was the anxiety score. Secondary mediation analysis was run by switching variables assigned to X and Me and if p_{Sobel} > 0.05 and T_{eff} < 50% for secondary mediation added the evidence that the mediation effects were directed. X= independent variable, Me = mediator, Y= dependent variable.

Mediation Variables				Step 1: X predicting Y		Step 2: X predicting Me		Step3: Me predicting Y		Step 3: Me (with X) predicting Y		Step 3: X (with Me) predicting Y		T_{eff}	p_{Sobel}
Y	Mediation	X	Me	c	p_c	a	p_a	b'	$p_{b'}$	b	p_b	c'	$p_{c'}$	%	
STAI-S	***Primary***	***Loss Resilience***	***age***	***-0.092***	***0.0000***	***0.139***	***0.0000***	***-0.434***	***0.0000***	***-0.434***	***0.0000***	***-0.031***	***0.0545***	***66.09***	***0.0000***
	Secondary	age	Loss Resilience	-0.438	0.0000	0.113	0.0000	-0.064	0.0003	-0.015	0.3446	-0.437	0.0000	0.39	0.3499
STAI-S	***Primary***	***Aversion TP***	***age***	***-0.052***	***0.0029***	***0.092***	***0.0000***	***-0.436***	***0.0000***	***-0.435***	***0.0000***	***-0.012***	***0.4338***	***76.24***	***0.0000***
	Secondary	age	Aversion TP	-0.438	0.0000	0.095	0.0000	-0.056	0.0015	-0.014	0.3675	-0.436	0.0000	0.31	0.3739
STAI-S	***Primary***	***Tradeoff Range***	***age***	***-0.153***	***0.0000***	***0.230***	***0.0000***	***-0.428***	***0.0000***	***-0.415***	***0.0000***	***-0.057***	***0.0006***	***62.41***	***0.0000***
	Secondary	age	Tradeoff Range	-0.428	0.0000	0.206	0.0000	-0.130	0.0000	-0.044	0.0089	-0.419	0.0000	2.10	0.0107
STAI-S	***Primary***	***Consistency Range***	***age***	***-0.070***	***0.0001***	***0.178***	***0.0000***	***-0.437***	***0.0000***	***-0.438***	***0.0000***	***0.008***	***0.6183***	***100.00***	***0.0000***
	Secondary	age	Consistency Range	-0.444	0.0000	0.177	0.0000	-0.068	0.0002	0.011	0.4860	-0.446	0.0000	0.00	0.4870
STAI-S	***Primary***	***Ante***	***loneliness***	***-0.057***	***0.0016***	***-0.119***	***0.0000***	***0.476***	***0.0000***	***0.476***	***0.0000***	***0.000***	***0.9844***	***100.00***	***0.0000***
	Secondary	loneliness	Ante	0.501	0.0000	-0.126	0.0000	-0.066	0.0002	-0.003	0.8550	0.501	0.0000	0.07	0.8551
STAI-S	***Primary***	***Insurance***	***loneliness***	***0.075***	***0.0000***	***0.109***	***0.0000***	***0.471***	***0.0000***	***0.468***	***0.0000***	***0.024***	***0.1357***	***68.13***	***0.0000***
	Secondary	loneliness	Insurance	0.497	0.0000	0.118	0.0000	0.082	0.0000	0.023	0.1361	0.494	0.0000	0.55	0.1458
STAI-S	***Primary***	***Total AR***	***loneliness***	***-0.070***	***0.0001***	***-0.079***	***0.0000***	***0.512***	***0.0000***	***0.510***	***0.0000***	***-0.029***	***0.0527***	***57.78***	***0.0000***
	Secondary	loneliness	Total AR	0.518	0.0000	-0.086	0.0000	-0.076	0.0000	-0.031	0.0385	0.515	0.0000	0.52	0.0564
STAI-S	***Primary***	***Consistency Range***	***loneliness***	***-0.050***	***0.0054***	***-0.102***	***0.0000***	***0.478***	***0.0000***	***0.478***	***0.0000***	***-0.001***	***0.9353***	***97.42***	***0.0000***
	Secondary	loneliness	Consistency Range	0.505	0.0000	-0.122	0.0000	-0.064	0.0004	-0.002	0.8879	0.504	0.0000	0.05	0.8879
STAI-S	***Primary***	***Consistency Range***	***employment***	***-0.055***	***0.0019***	***0.155***	***0.0000***	***-0.290***	***0.0000***	***-0.289***	***0.0000***	***-0.011***	***0.5366***	***80.73***	***0.0000***
	Secondary	employment	Consistency Range	-0.288	0.0000	0.095	0.0000	-0.041	0.0221	-0.014	0.4149	-0.287	0.0000	0.47	0.4204
STAI-S	***Primary***	***-***	***income***												
	Secondary	income	-												

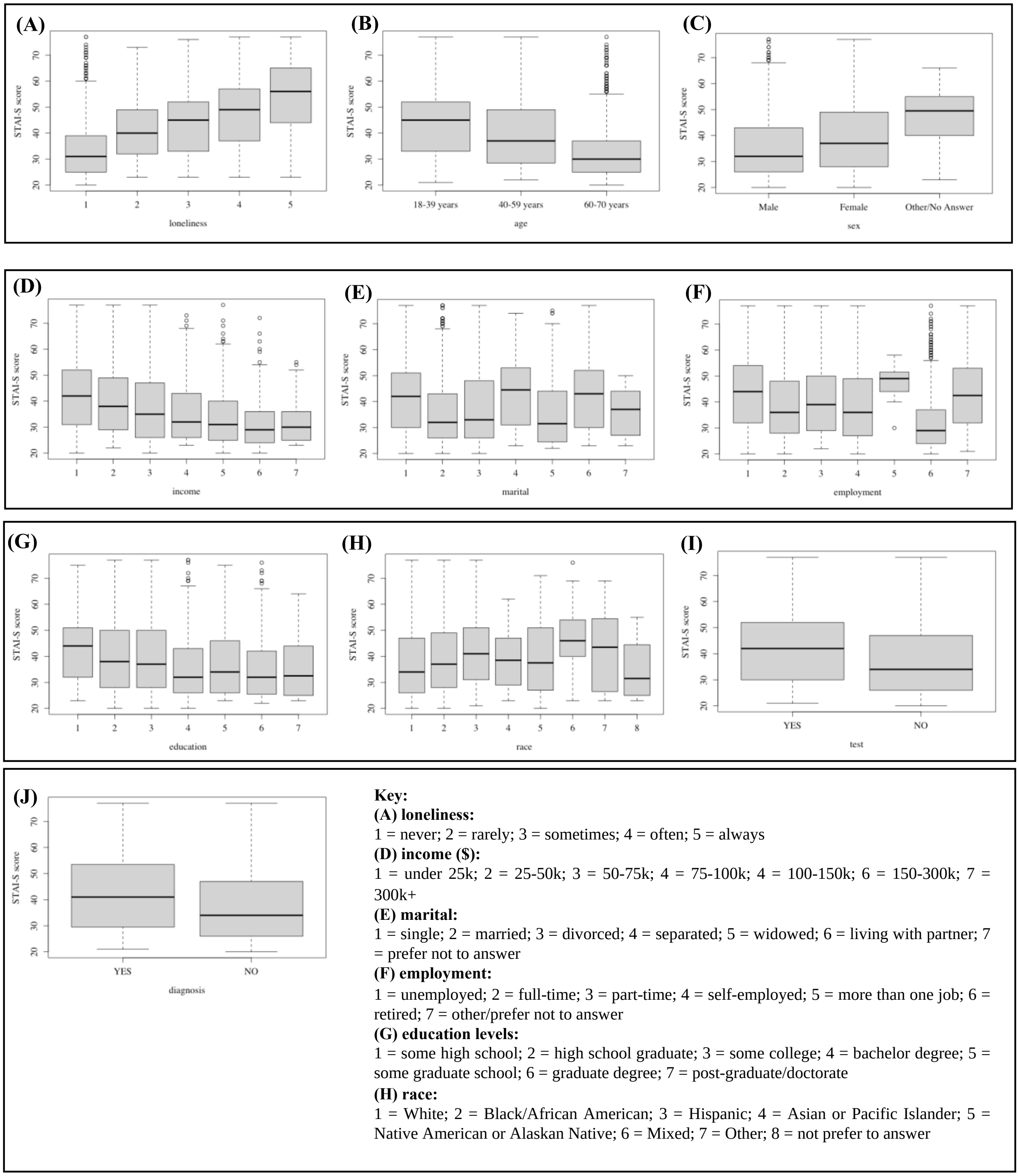

Supplementary Figure 3. Boxplots of STAI-S/anxiety scores with respect to the levels of contextual variables. The contextual variables included: **(A)** perceived loneliness in the past one month (loneliness), **(B)** age, **(C)** gender assigned at birth (sex), **(D)** annual household income (income), **(E)** marital status (marital), **(F)** employment status (employment), **(E)** level of education (edu), **(H)** ethnicity (race), **(I)** if the participant had ever tested positive for COVID-19 (test) and **(J)** if the participant was ever diagnosed by a clinician with COVID-19 (diagnosis). The key contains the explanation of what each level on the x-axis corresponds to.

Reference:

1. S. Bari, N. L. Vike, K. Stetsiv, S. Woodward, S. Lalvani, L. Stefanopoulos, B. W. Kim, N. Maglaveras, H. C. Breiter, A. K. Katsaggelos, The Prevalence of Psychotic Symptoms, Violent Ideation, and Disruptive Behavior in a Population With SARS-CoV-2 Infection: Preliminary Study. *JMIR Form Res 2022;6(8):e36444 https://formative.jmir.org/2022/8/e36444* **6**, e36444 (2022).
2. S. Woodward, S. Bari, N. Vike, S. Lalvani, K. Stetsiv, B. W. Kim, L. Stefanopoulos, N. Maglaveras, H. Breiter, A. Katsaggelos, Anxiety, Post-COVID-Syndrome-related depression, and suicidal thinking and behavior in COVID-19 survivors. *JMIR Form Res* (2022), doi:10.2196/36656.
3. N. L. Vike, S. Bari, K. Stetsiv, S. Woodward, S. Lalvani, L. Stefanopoulos, B. W. Kim, N. Maglaveras, A. K. Katsaggelos, H. C. Breiter, The Relationship Between a History of High-risk and Destructive Behaviors and COVID-19 Infection: Preliminary Study. *JMIR Form Res 2023;7:e40821 https://formative.jmir.org/2023/1/e40821* **7**, e40821 (2023).
4. C. D. Spielberger, Manual for the State-Trait Anxiety Inventory. *Palo Alto, CA: Consulting Psychologists* (1983).
5. B. W. Kim, D. N. Kennedy, J. Lehár, M. J. Lee, A. J. Blood, S. Lee, R. H. Perlis, J. W. Smoller, R. Morris, M. Fava, H. C. Breiter, Recurrent, robust and scalable patterns underlie human approach and avoidance. *PLoS One* **5**, e10613 (2010).
6. V. Viswanathan, J. P. Sheppard, B. W. Kim, C. L. Plantz, H. Ying, M. J. Lee, K. Raman, F. J. Mulhern, M. P. Block, B. Calder, S. Lee, D. T. Mortensen, A. J. Blood, H. C. Breiter, A quantitative relationship between signal detection in attention and approach/avoidance behavior. *Front Psychol* **8**, 122 (2017).
7. S. L. Livengood, J. P. Sheppard, B. W. Kim, E. C. Malthouse, J. E. Bourne, A. E. Barlow, M. J. Lee, V. Marin, K. P. O’Connor, J. G. Csernansky, M. P. Block, A. J. Blood, H. C. Breiter, Keypress-based musical preference is both individual and lawful. *Front Neurosci* **11**, 136 (2017).
8. V. Viswanathan, S. Lee, J. M. Gilman, B. W. Kim, N. Lee, L. Chamberlain, S. L. Livengood, K. Raman, M. J. Lee, J. Kuster, D. B. Stern, B. Calder, F. J. Mulhern, A. J. Blood, H. C. Breiter, Age-related striatal BOLD changes without changes in behavioral loss aversion. *Front Hum Neurosci* **9**, 1–12 (2015).
9. G. P. Gasic, J. W. Smoller, R. H. Perlis, M. Sun, S. Lee, B. W. Kim, M. J. Lee, D. J. Holt, A. J. Blood, N. Makris, D. K. Kennedy, R. D. Hoge, J. Calhoun, M. Fava, J. F. Gusella, H. C. Breiter, BDNF, relative preference, and reward circuitry responses to emotional communication. *American Journal of Medical Genetics, Part B: Neuropsychiatric Genetics* **150**, 762–781 (2009).
10. R. H. Perlis, D. J. Holt, J. W. Smoller, A. J. Blood, S. Lee, B. W. Kim, M. J. Lee, M. Sun, N. Makris, D. K. Kennedy, K. Rooney, D. D. Dougherty, R. Hoge, J. F. Rosenbaum, M. Fava, J. Gusella, G. P. Gasic, H. C. Breiter, Association of a polymorphism near CREB1 with differential aversion processing in the insula of healthy participants. *Arch Gen Psychiatry* **65**, 882–892 (2008).
11. I. Aharon, N. Etcoff, D. Ariely, C. F. Chabris, E. O’Connor, H. C. Breiter, Beautiful faces have variable reward value: fMRI and behavioral evidence. *Neuron* **32**, 537–551 (2001).
12. N. Makris, M. Oscar-Berman, S. K. Jaffin, S. M. Hodge, D. N. Kennedy, V. S. Caviness, K. Marinkovic, H. C. Breiter, G. P. Gasic, G. J. Harris, Decreased Volume of the Brain Reward System in Alcoholism. *Biol Psychiatry* **64**, 192–202 (2008).

13. M. M. Strauss, N. Makris, I. Aharon, M. G. Vangel, J. Goodman, D. N. Kennedy, G. P. Gasic, H. C. Breiter, fMRI of sensitization to angry faces. *Neuroimage* **26**, 389–413 (2005).
14. E. A. Azcona, B.-W. Kim, N. L. Vike, S. Bari, S. Lalvani, L. Stefanopoulos, S. Woodward, M. Block, A. K. Katsaggelos, H. C. Breiter, Discrete, recurrent, and scalable patterns in human judgement underlie affective picture ratings. (2022), doi:10.48550/arxiv.2203.06448.
15. H. Markowitz, Portfolio Selection. *J Finance* **7**, 77–91 (1952).
