# Supplementary material for: A novel approach to anxiety level prediction using small sets of judgment and survey variables
Source: Npj Ment Health Res. 2024 Jun 18;3:29. doi: 10.1038/s44184-024-00074-x (PMC11189415; doi:10.1038/s44184-024-00074-x)
Supplement: Supplementary file 3 — Supplemental Appendix 2 [file 44184_2024_74_MOESM3_ESM.docx]

# Use RF/bRF to predict low/high class for STAI-S with 15judgmentvariables+demographics

# Code written by: Sumra Bari

rm(list = ls()) # clear all objects

graphics.off() # close graphics windows

# Loading package

require(readxl)

library(randomForest)

library(caret)

library(ROCR)

# Load the data -------------------------------------------------------

# set the working directory

# path to the folder where script is located

path_to_folder = dirname(rstudioapi::getSourceEditorContext()$path)

path_to_file = file.path(path_to_folder,'..','4019dataset',

'Data_4019.xlsx',

fsep = '/')

mydata = read_excel(path_to_file,sheet = 1)

mydata = mydata[mydata$Keep==1,]

# define the variables and class labels --------------------------------

mydata$age = mydata$age+16

mydata$edu_years = mydata$edu_years+7

RPT = mydata[,2:16]

# define low and high class for STAI-------------------

# define threshold to divide the data into low and high classes

Th = 35

Class = c()

Class[mydata$`STAI-Ssum`< Th]='L'

Class[mydata$`STAI-Ssum`>=Th]='H'

cat ('Percentage class L \n')

print(100*sum(Class == 'L')/length(Class))

cat ('Percentage class H \n')

print(100*sum(Class == 'H')/length(Class))

# add demographics to RPT data frame ------------------------------------

RPT$age = mydata$age

RPT$edu_years = mydata$edu_years

RPT$race = factor(mydata$race)

RPT$income = factor(mydata$income)

RPT$edu = factor(mydata$edu_level)

RPT$employment = factor(mydata$employment)

RPT$marital = factor(mydata$marital)

RPT$sex = factor(mydata$gender)

RPT$loneliness = factor(mydata$L1)

RPT$test =factor(mydata$test)

RPT$diagnosis = factor (mydata$diagnosis)

# place the class labels at the end of data frame

RPT$Class = factor(Class)

# divide the data in to train and test splits ---------------------------

ind <- sample(2, nrow(RPT), replace = TRUE, prob = c(0.7, 0.3))

train <- RPT[ind==1,]

test <- RPT[ind==2,]

# get the number of minimum number of samples out of the two classes -----

nmin = min(sum(train$Class == 'L'),sum(train$Class == 'H'))

# Fitting Random Forest to the train dataset --------------------

classifier_RF = randomForest(x = train[-length(RPT)],

y = train$Class,

# Tell randomForest to sample by strata. Here,

# that means within each class

strata = train$Class, #------------for bRF

# Now specify that the number of samples selected

# within each class should be the same

sampsize = rep(nmin,2), #----------for bRF

#importance = TRUE, # Gives MeanDecAccuracy measure

ntree = 1000,

proximity = TRUE)

#classifier_RF <- randomForest(Class~., data=train, proximity=TRUE)

print(classifier_RF)

# Predicting the Test set ------------------------------------

y_pred = predict(classifier_RF, newdata = test[-length(RPT)])

cat('-------------FOR TEST SET-------------------\n')

print(confusionMatrix(y_pred, test$Class))

# Get the ROC AUC measure for test set-------------------------------------------

# for test set

rf_p_test <- as.numeric(predict(classifier_RF, test[-length(RPT)], type="response"))

rf_p_test[rf_p_test ==2]=0

rf_pr_test <- prediction(rf_p_test, test$Class,label.ordering = c('L','H'))

rf_pl_test <- performance(rf_pr_test , measure = "tpr", x.measure = "fpr")

plot(rf_pl_test, col=rainbow(10))

r_auc_test<- performance(rf_pr_test ,measure = "auc")@y.values[[1]]

cat("ROC AUC for test set:\n")

print(r_auc_test)

# Plotting model-------------------------------------------------------------------

plot(classifier_RF)

# Importance plot------------------------------------------------------------------

importance(classifier_RF,2)

# Variable importance plot

#increase the size of the labels and make labels Times New Roman

par(cex.axis = 1.25,cex.lab = 1.25,cex.main =1.25,family="serif", font=1)

varImpPlot(classifier_RF, sort = TRUE, scale = FALSE, n.var = length(RPT)-1,

main = 'Feature Importance (in descending order)')

# Dimension plot-showing a 2-d projection of distances between samples----------

MDSplot(classifier_RF,train$Class,palette = c(1,2),pch = 1)

legend('topright',pch = 1,

col=c('black','red'),

c('High','Low'))

# Relative Importance Tables ---------------------------------------------------

RI = importance(classifier_RF,2)/sum(importance(classifier_RF,2))
